# Novel Treatment of Chronic Graft-Versus-Host Disease in Mice Using the ER Stress Reducer 4-Phenylbutyric Acid

**DOI:** 10.1038/srep41939

**Published:** 2017-02-06

**Authors:** Shin Mukai, Yoko Ogawa, Fumihiko Urano, Chie Kudo-Saito, Yutaka Kawakami, Kazuo Tsubota

**Affiliations:** 1 Deaprtment of Ophthalmology, Keio University School of Medicine, 35 Shinanomachi, Shinjuku-ku, Tokyo, 160-8582, Japan; 2 Institute for Advanced Medical Research, Keio University School of Medicine, 35 Shinanomachi, Shinjuku-ku, Tokyo, 160-8582, Japan; 3 Department of Medicine, Division of Endocrinology, Metabolism, and Lipid Research, and Department of Pathology and Immunology, Washington University School of Medicine, 660 S. Euclid Ave Campus Box 8127, St. Louis, MO 63110, USA.

## Abstract

Chronic graft-versus-host disease (cGVHD) is a notorious complication of allogeneic hematopoietic stem cell transplantation and causes disabling systemic inflammation and fibrosis. In this novel study, we focused on a relationship between endoplasmic reticulum (ER) stress and cGVHD, and aimed to create effective treatment of cGVHD. A series of experiments were conducted using a mouse model of cGVHD. Our data suggested (1) that ER stress was elevated in organs affected by cGVHD and (2) that 4-phenylbutyric acid (PBA) could reduce cGVHD-induced ER stress and thereby alleviate systemic inflammation and fibrosis. Because fibroblasts are thought to be implicated in cGVHD-elicited fibrosis and because macrophages are reported to play a role in the development of cGVHD, we investigated cGVHD-triggered ER stress in fibroblasts and macrophages. Our investigation demonstrated (1) that indicators for ER stress and activation markers for fibroblasts were elevated in cGVHD-affected lacrimal gland fibroblasts and (2) that they could be reduced by PBA. Our work also indicated that splenic macrophages from PBA-dosed mice exhibited the lower levels of ER stress and M2 macrophage markers than those from cGVHD-affected mice. Collectively, this study suggests that the reduction of ER stress utilizing PBA can be a clinically translatable method to treat systemic cGVHD.

Chronic graft-versus-host disease (cGVHD) is a disabling complication after allogeneic hematopoietic stem cell transplantation (HSCT) and has a highly negative impact on patients’ quality of life. Unfortunately, there has been painfully slow progress in developing effective treatment of cGVHD thus far. Acute GVHD (aGVHD) has been studied intensively, and there is a growing understanding of aGVHD. In contrast, sparse information about mechanisms of cGVHD has been provided. Although immunosuppressant such as cyclosporine has been utilized to cure cGVHD in medical practice, it has been only partially effective for cGVHD. Most recently, several strategies to treat cGVHD have been reported^1^,^2^,^3^,^4^. However, there is still vast scope for improvement in the treatment of cGVHD. aGVHD is initiated by preparative regimens which induce host tissue damage, proinflammatory cytokine release and chemokine production^5^,^6^,^7^,^8^. Donor T-cells subsequently recognize host alloantigens as foreign bodies and thereby attack recipient cells^9^,^10^. The two types of GVHD should be classified by clinical manifestations, not by time after HSCT^11^. However, aGVHD typically appears within 100 days after HSCT, and cGVHD usually manifests 6 months or later after HSCT and resembles autoimmune diseases^12^,^13^. While cGVHD is largely unexplored, it has been reported that cellular senescence is detrimentally involved in ocular cGVHD and that cGVHD can be regarded as an age-associated disease^14^. In clinical settings, gray eyebrows, skin wrinkles and conjunctival cancer are observed, which suggests that recipient cells as well as donor immune cells are senescent in cGVHD^14^,^15^.

Literature precedent indicates that endoplasmic reticulum (ER) stress plays a contributory role in chronic inflammation and age-related diseases^16^,^17^. The ER is a cellular organelle essential for cells to function properly. When proteins are synthesized in the ER, they need to be folded adequately and ER chaperones assist the protein folding^18^. However, hypoxia, calcium ion depletion, oxidative injury, viral infections and inflammatory cytokines prevent the ER from performing its normal protein folding^19^,^20^. When unfolded and misfolded proteins are accumulated in the ER, it causes ER stress and the following three transmembrane proteins are consequently activated to initiate the unfolded protein response (UPR): inositol requiring (IRE) 1α, PKR-like ER kinase (PERK) and activating transcription factor (ATF) 6α^18^. However, if the UPR is prolonged or unsuccessful, inflammatory and apoptotic pathways are activated^18^. Hence, the unsuccessful UPR leads to the expression of (1) the proinflammatory molecules thioredoxin interaction protein (TXNIP) and transcription factor nuclear factor kappa-light-chain-enhancer of activated B cells (NF-κB), and (2) the apoptotic protein C/EBP homologous protein (CHOP)^21^,^22^,^23^,^24^,^25^,^26^.

In this study, we aimed to devise effective treatment of cGVHD. Based on the previous findings, we envisaged (1) that ER stress would be increased in cGVHD-impaired organs and (2) that mitigation of ER stress could be effective treatment of cGVHD. To achieve this goal, we used 4-phenylbutyric acid (PBA) as an ER stress reducer^27^. (Supplementary Figure 1A) PBA is commercially available and can be an inexpensive starting material in organic synthesis^28^. In clinical settings, PBA is approved by FDA and has been utilized to cure urea cycle disorders^27^. Furthermore, several articles demonstrate that PBA can be effective for chronic inflammation and age-related diseases such as type 2 diabetes and obesity-caused chronic inflammation through mitigation of ER stress^29^,^30^. Hence, it was envisioned that PBA could be a safe and efficacious drug for cGVHD, in which systemic inflammation and fibrosis are highly problematic. Herein, we report the world-first strategy to treat cGVHD using the ER stress reducer PBA.

## Results

### Elevation of ER stress markers in cGVHD-affected organs

**Figure 1:**
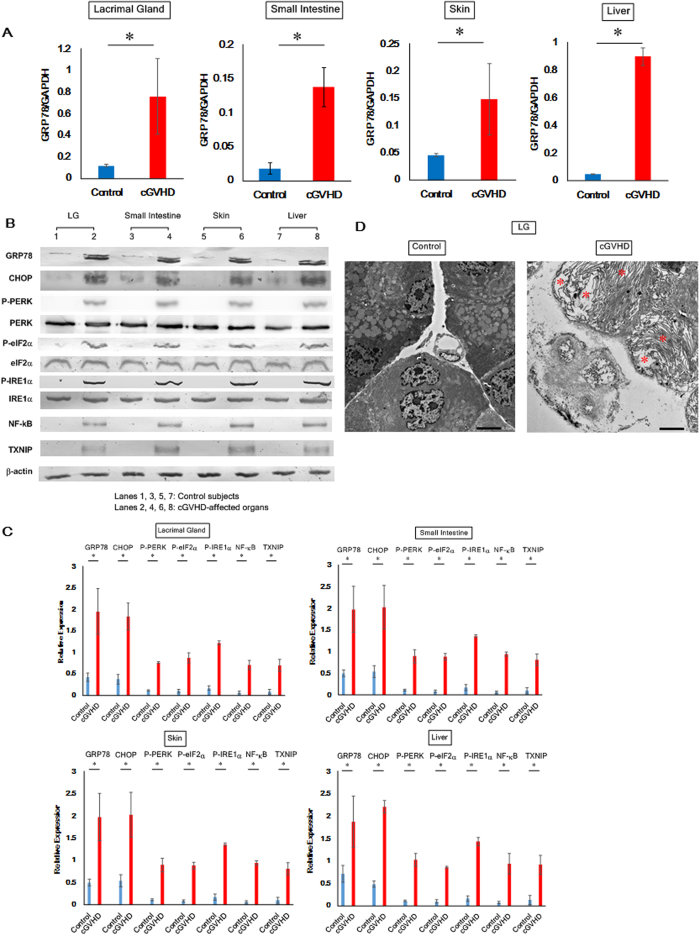
ER stress markers in cGVHD target organs. (**A**) qPCR analysis of GRP78 in cGVHD-affected organs (red) and control subjects (blue). Data from one of two similar experiments are shown. The data are presented as means, ±SD, Control: n = 5, cGVHD: n = 4–5 *P < 0.05. (**B**) Immunoblot analysis of ER stress indicators and the associated inflammatory molecules was carried out. (Lanes 1, 3, 5, 7: Syngeneic control subjects, Lanes 2, 4, 6, 8: cGVHD-impaired organs) Cropped blots are displayed. (**C**) The corresponding quantitative analysis of the protein bands was conducted. cGVHD-affected organs (red) and control subjects (blue). Data from one of two similar experiments are shown. The data are presented as means, ±SD, Control: n = 4, cGVHD: n = 4 *P < 0.05. (**D**) Electron micrographs of epithelial cells in the cGVHD-affected lacrimal gland and those in its syngeneic control counterpart. The pictures were taken at 2000x magnification, and the scale bar is 5 μm. In the image of cGVHD-affected lacrimal gland epithelia, asterisks are placed where the ER was expanded due to the accumulation of proteins.

The initial venture toward our overarching aim was to investigate whether ER stress was elevated in cGVHD-affected organs by measuring ER stress markers. Real-time quantitative PCR (qPCR) and immunoblot analysis suggested (1) that the ER stress indicators GRP78, CHOP, p-PERK, p-eIF2α, and p-IRE1α were increased in murine organs affected by cGVHD compared with control subjects and (2) that accordingly the following two inflammatory molecules were activated and/or augmented in the cGVHD-impaired organs: NF-κB and TXNIP (Fig. 1A–C). As evidenced by electron micrographs, in cGVHD-impaired lacrimal gland epithelia, accumulated misfolded/unfolded proteins caused the ER to expand (Fig. 1D). Conversely, the ER in the controls seemed intact (Fig. 1D). These observations are indicative of the elevation of ER stress in cGVHD-affected organs.

### Deactivation of the ER stress-induced inflammatory pathways utilizing PBA

**Figure 2:**
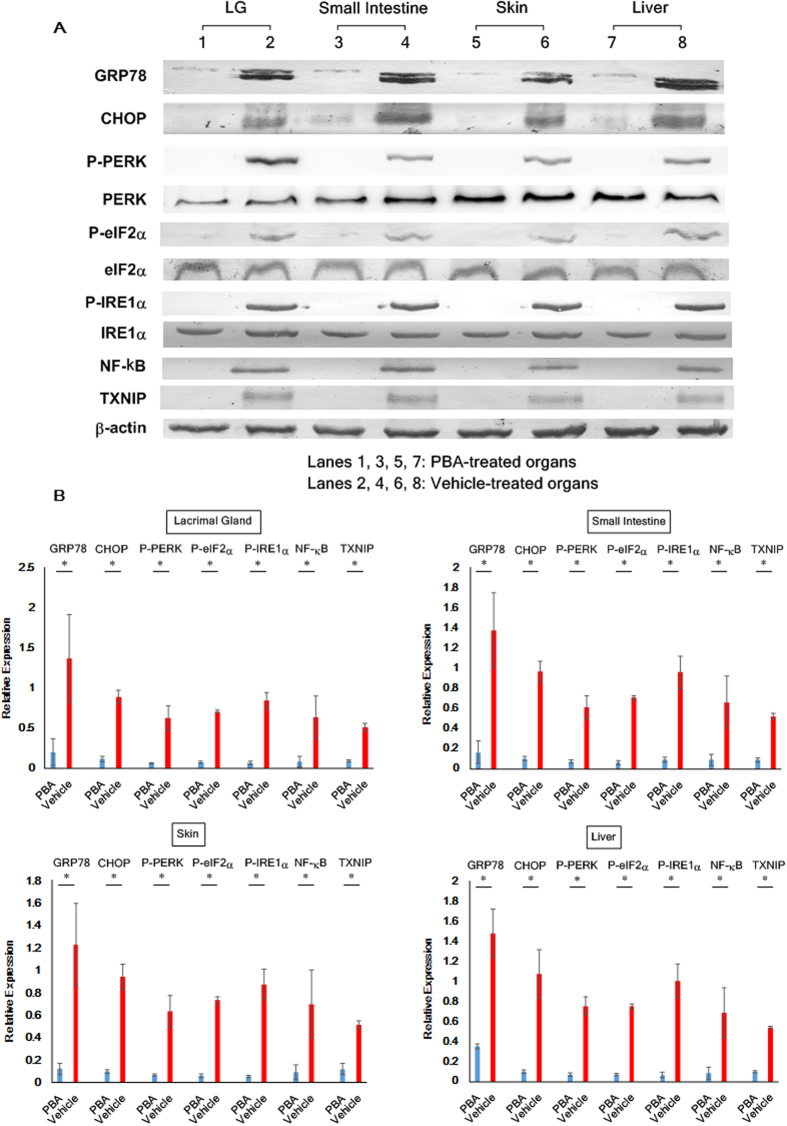
Suppression of cGVHD-caused ER stress by PBA. (**A**) Immunoblot assays were performed to examine the protein levels of ER stress markers and the associated inflammatory molecules in the cGVHD target organs. (Lanes 1, 3, 5, 7: PBA-medicated organs. Lanes 2, 4, 6, 8: Vehicle-medicated organs) Cropped blots are displayed. (**B**) The target proteins in each organ were subsequently quantified by densitometry. PBA-treated organs (blue) and vehicle-treated organs (red). Data from one of two similar experiments are shown. The data are presented as means, ±SD, PBA: n = 4, Vehicle: n = 4, *P < 0.05.

Once we found that ER stress was increased in the organs impaired by cGVHD, our next attempt was to treat cGVHD through reduction of ER stress by the use of PBA. As depicted in the Methods section, we administered PBA or the solvent-vehicle to mice which underwent allogeneic BMT. Immunoblot analysis indicated that organs collected from the PBA-treated mice exhibited the lower protein levels of GRP78, CHOP, p-PERK, p-eIF2α, and p-IRE1αcompared with those from the vehicle-treated mice (Fig. 2A,B; Supplementary Figure 2A,B). Consequently, the associated proinflammatory molecules NF-κB and TXNIP were subdued in the organs treated with PBA compared to their vehicle-treated counterparts (Fig. 2A,B; Supplementary Figure 2A,B).

### Histological observations of cGVHD target organs

**Figure 3:**
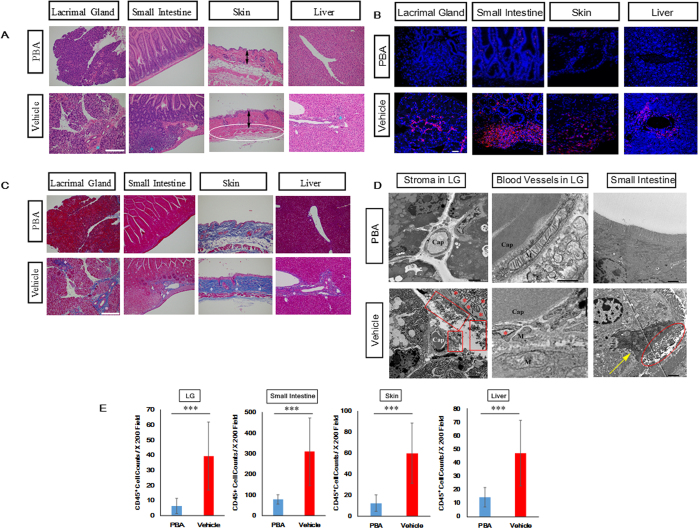
Mitigation of cGVHD-elicited systemic inflammation and fibrosis using the ER stress mitigator PBA. (**A**) HE pictures of the PBA-treated organs and those treated with the solvent-vehicle. The images were taken at 200x magnification, and the scale bar is 200 μm. Severely inflamed portions are shown with blue asterisks. In the picture of the vehicle-medicated skin, an ellipse is placed where the fatty tissues were lost. The thickness of the PBA- and vehicle-treated skin was also displayed with arrows. (**B**) Immunostaining for the generic leukocyte marker CD45 in the PBA-medicated tissues and the vehicle-medicated ones. Cell cytosol and nuclei are stained red and blue, respectively. The images were taken at 200x magnification, and the scale bar is 20 μm. (**C**) Mallory’s staining for the PBA-injected organs and their vehicle-injected counterparts. The photographs were taken at 200x magnification, and the scale bar is 200 μm. Aberrantly fibrotic areas are shown with white asterisks. (**D**) Electron micrographs of the PBA-treated lacrimal glands and small intestine, and their vehicle-treated counterparts. The pictures of stroma of the lacrimal glands (left) and epithelial cells of the small intestine (right) were taken at 2000x magnification, and the scale bar is 5 μm. The photographs of blood vessels in the lacrimal glands were taken at 15000x magnification, and the scale bar is 500 nm (middle). Cap: Capillary, M: Mitochondrion. In the pictures of the vehicle-medicated lacrimal glands, asterisks are placed where the ER is expanded due to the accumulation of proteins, and cell debris is shown with a rectangle. In the image of the vehicle-treated small intestine, an ellipse is placed where microvilli were demolished, and damaged tissues are indicated with an arrow. (**E**) The density of CD45 positive cells in the PBA-treated organs (blue) and the vehicle-treated ones (red). Data from one of two similar experiments are shown. The data are presented as means, ±SD, PBA: n = 3, Vehicle n = 3, *P < 0.05, **P < 0.01, ***P < 0.001.

HE and Mallory pictures of cGVHD-susceptible organs indicate that the reduction of ER stress using PBA can be efficacious treatment of cGVHD (Fig. 3). As judged by the HE pictures, inflammatory cell infiltration was repressed in the PBA-medicated organs compared with their vehicle-medicated counterparts (Fig. 3A; Supplementary Figure 3A). Particularly, (1) the intestinal villi of the PBA-treated small and large intestines seemed intact in contrast to those treated with the solvent-vehicle, (2) the vehicle-treated skin was thickened, lost the fatty tissues and had the elevated density of collagen bundles by contrast with the PBA-treated skin, (3) meibomian glands in the vehicle-medicated eyes were decreased and shrunk compared with their PBA-medicated counterparts and (4) the thinning and damage in vehicle-injected conjunctival epithelia were observed in contrast to their PBA-injected counterparts, which suggested that a symblepharon could be prevented by systemic injection of PBA (Fig. 3A, Supplementary Figure 3A). As indicated by the immunostaining and subsequent counting of CD45^+^ cells, the number of inflammatory cells in the PBA-treated organs was considerably lower than that in their vehicle-treated counterparts (Fig. 3B,E; Supplementary Figure 3B,D). These observations are also indicative of the suppression of immune cell migration and proliferation in the PBA-treated organs. Furthermore, systemic fibrosis is one of the most serious problems in cGVHD^31^. Mallory’s staining revealed that cGVHD-induced fibrosis was substantially subdued in the PBA-medicated organs compared with the vehicle-medicated ones (Fig. 3C; Supplementary Figure 3C). Electron micrographs of the lacrimal glands indicate that in the case where the lacrimal glands were treated with the solvent-vehicle, (1) the ER of the epithelial and endothelial cells were expanded due to the accumulation of unfolded/misfolded proteins, (2) there was a large amount of cell debris in the stroma and (3) mitochondria in the epithelial and endothelial cells were damaged (Fig. 3D). Electron micrographic analysis of the small intestine also demonstrated that when the small intestine was treated with the solvent-vehicle, its microvilli were demolished and the neighboring tissues were damaged (Fig. 3D). These histological findings were not observed in the PBA-treated lacrimal glands or small intestine. In addition, PAS staining revealed (1) that goblet cells in the PBA-injected small intestine and conjunctival epithelia outnumbered those in their vehicle-injected counterparts and (2) that the structures of intestinal and conjunctival mucosal membranes were retained by systemic injection of PBA (Supplementary Figure 4A,B). As judged by all the histological observations, PBA can alleviate systemic inflammation and fibrosis elicited by cGVHD-related ER stress.

### Reduction of inflammatory and pro-fibrotic mediators by the use of PBA

**Figure 4:**
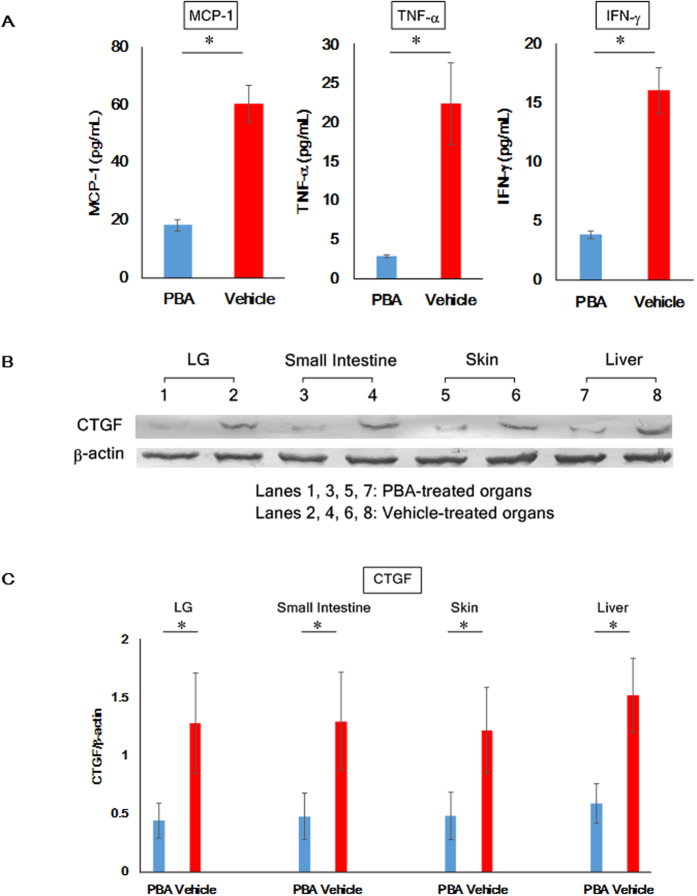
Reduction of indicators for inflammation and fibrosis utilizing the ER stress alleviator PBA. (**A**) ELISA was carried out to measure the inflammatory indicators MCP-1, TNF-α and IFN-γ in sera collected from PBA-treated mice (blue) and their vehicle-treated counterparts (red) 28 days after BMT. Data from one of two similar experiments are shown. The data are presented as means ± SD, PBA: n = 4, Vehicle n = 4, *P < 0.05 (**B**) Immunoblot analysis of the fibrotic marker CTGF was conducted. (Lanes 1, 3, 5, 7: PBA-treated organs, Lanes 2, 4, 6, 8: vehicle-treated organs) Cropped blots are displayed. (**C**) CTGF in each organ was subsequently quantified by densitometry. PBA-treated organs (blue) and vehicle-treated organs (red). Data from one of two similar experiments are shown. The data are presented as means, ± SD, PBA: n = 4, Vehicle n = 4, *P < 0.05.

To examine more closely whether PBA could mitigate cGVHD-caused systemic inflammation, ELISA was conducted to measure the levels of the inflammatory molecules MCP-1, TNF-α and IFN-γ in sera collected from the PBA- and vehicle-injected mice. The sera from the PBA-medicated mice exhibited the lower protein levels of MCP-1, TNF-α and IFN-γ in comparison to those from their vehicle-medicated counterparts. (Figure 4A) Moreover, we performed more meticulous investigation into the alleviation of cGVHD-triggered systemic fibrosis, and immunoblot analysis revealed that the pro-fibrotic mediator connective tissue growth factor (CTGF) was overexpressed in the vehicle-treated organs, yet it was not observed in those treated with PBA (Fig. 4B,C; Supplementary Figure 5A,B). These data support that PBA can repress cGVHD-induced ER stress and consequently mitigate systemic inflammation and fibrosis.

### Correlation between ER stress and dysfunctional fibroblasts

**Figure 5:**
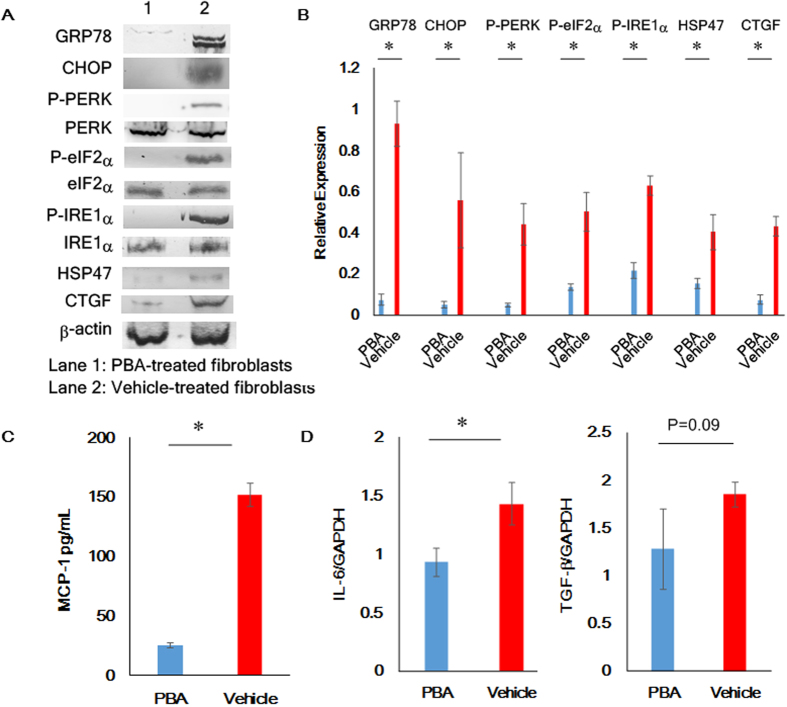
Alleviation of cGVHD-triggered ER stress in lacrimal gland fibroblasts utilizing the ER stress attenuator PBA. (**A**) Immunoblot analysis of ER stress, activation and fibrotic markers in murine lacrimal gland fibroblasts was carried out. (Lane 1: Fibroblasts from the PBA-treated lacrimal glands, Lane 2: Fibroblasts from the vehicle-treated lacrimal glands) Cropped blots are displayed. (**B**) The corresponding quantitative analysis of the protein bands was conducted. PBA-treated fibroblasts (blue) and vehicle-treated fibroblasts (red). Data from one of two similar experiments are shown. The data are presented as means, ±SD, PBA: n = 4, Vehicle n = 4, *P < 0.05. (**C**) ELISA to measure the protein levels of MCP-1 produced by the PBA- and vehicle-treated fibroblasts. PBA-treated fibroblasts (blue) and vehicle-treated fibroblasts (red). Data from one of two similar experiments are shown. The data are presented as means, ± SD, PBA: n = 4, Vehicle n = 4, *P < 0.05. (**D**) qPCR analysis of IL-6 and TGF-β in the PBA-medicated fibroblasts and their vehicle-medicated counterparts. PBA-treated fibroblasts (blue) and vehicle-treated fibroblasts (red). Data from one of two similar experiments are shown. The values were means, ±SD, PBA: n = 4, Vehicle n = 4, *P < 0.05.

Systemic fibrosis arising from cGVHD is a serious problem and causes multiple organ failure^31^. Although the mechanisms of cGVHD-triggered fibrosis still need to be elucidated, fibroblasts are conceivably implicated in the development of fibrosis^32^. Several biological signals stimulate fibroblasts so that they can play an integral role in several biological processes such as wound healing^33^,^34^. However, if fibroblasts are activated in an uncontrolled manner, they produce abnormal collagen bundles and thereby induce severe fibrosis although there is little information about what causes their abnormality^33^,^34^. Hence, we strived to unravel a relationship between cGVHD-elicited ER stress and dysfunctional fibroblasts. To accomplish this goal, we cultured fibroblasts from the PBA- and vehicle-treated murine lacrimal glands as described in the Methods section^35^. As confirmed by immunoblot assays, the cultured lacrimal gland fibroblasts (expressing α-SMA) were not contaminated with epithelial cells (cytokeratin positive cells) (Supplementary Figure 13), and the following experiments were carried out by the use of sufficiently pure fibroblasts. Immunoblot analysis of the cultured fibroblasts suggested that GRP78, CHOP, phosphorylated PERK, phosphorylated eIF2α and phosphorylated IRE1α were greatly suppressed by treatment of the mice with PBA in comparison to the solvent-vehicle (Fig. 5A). Accordingly, (1) the protein levels of HSP47 and CTGF, markers for fibroblast activation and fibrosis, respectively, (2) the production of MCP-1 and (3) the mRNA level of IL-6 were reduced in the PBA-treated fibroblasts compared with their vehicle-treated counterparts (Fig. 5A–D).

### Link between ER stress and the polarization of macrophages

**Figure 6:**
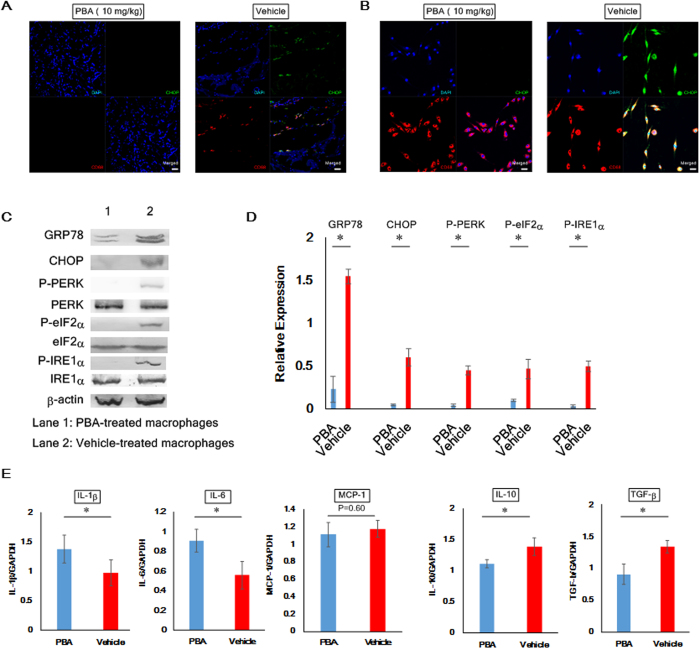
Mitigation of cGVHD-caused ER stress in macrophages by the use of PBA. (**A**) Immunofluorescence images of the PBA- and vehicle-injected lacrimal glands. Macrophages and CHOP are stained red and green, respectively. The pictures were taken at 200x magnification, and the scale bar is 20 μm. (**B**) Immunofluorescence images of cultured splenic macrophages from the PBA- and the vehicle-dosed mice. Macrophages and CHOP are stained red and green, respectively. The photographs were taken at 200x magnification, and the scale bar is 20 μm. (**C**) Immunoblot analysis of ER stress markers in murine splenic macrophages. (Lane 1: Splenic macrophages from PBA-dosed mice, Lane 2: Splenic macrophages from vehicle-dosed mice) Cropped blots are displayed. (**D**) The corresponding quantitative analysis of each protein band. PBA-treated macrophages (blue) and vehicle-treated macrophages (red). Data from one of two similar experiments are shown. The data are shown as means, ±SD, PBA: n = 4, Vehicle n = 4, *P < 0.05. (**E**) qPCR analysis of markers for M1 and M2 macrophages from the spleens. PBA-treated macrophages (blue) and vehicle-treated macrophages (red). Data from one of two similar experiments are shown. The data are presented as means, ±SD, PBA: n = 4–5, Vehicle n = 4–5, *P < 0.05.

Macrophages serve a crucial role in immune responses and inflammation, and an earlier article indicates that senescent macrophages are detrimentally linked with the pathogenic process of cGVHD^14^. Hence, we scrutinized a relationship between cGVHD-induced ER stress and malfunctioning macrophages. In the first instance, immunostaining was conducted to investigate whether macrophages in the murine lacrimal glands expressed the ER stress indicator CHOP. As judged by fluorescence images of the lacrimal glands, when mice were medicated with the solvent-vehicle, macrophages expressing CHOP migrated to the tissues (Fig. 6A). In contrast, no such macrophages were observed in the lacrimal glands collected from the PBA-medicated mice (Fig. 6A). Our subsequent endeavors were to gain greater insights into ER stress in cGVHD-affected macrophages. Toward this end, we cultured splenic macrophages from PBA- and vehicle-injected mice as depicted in the Methods section^36^. As evidenced by immunoblot analysis, the induced macrophages (expressing CD68) were not adulterated with T-cells (CD3^+^ cells) or B-cells (CD19^+^ and/or CD20^+^ cells) (Supplementary Figure 14), and the following results were gleaned utilizing highly pure macrophages. Immunohistochemistry revealed that the vehicle-medicated splenic macrophages expressed CHOP in contrast to the PBA-medicated ones (Fig. 6B). Immunoblot analysis of the macrophages also indicated that in the case where they were treated with PBA, the ER stress markers GRP78, CHOP, phosphorylated PERK, phosphorylated eIF2α and phosphorylated IRE1α were substantially repressed compared with the solvent-vehicle (Fig. 6C,D). We then conducted qPCR to examine the gene expression of (1) the M1 macrophage markers IL-1β, IL-6 and MCP-1 and (2) the M2 macrophage markers TGF-β and IL-10 in the splenic macrophages^37^. As demonstrated by the data, the mRNA expression of TGF-β and IL-10 was increased in the vehicle-treated macrophages in comparison to the PBA-treated ones (Fig. 6E). Conversely, when macrophages were treated with PBA, they showed the higher gene expression of IL-1β and IL-6 than those treated with the solvent-vehicle (Fig. 6E). These outcomes are indicative of the connection between cGVHD-elicited ER stress in macrophages and their differentiation into an alternatively activated phenotype.

## Discussion

**Figure 7:**
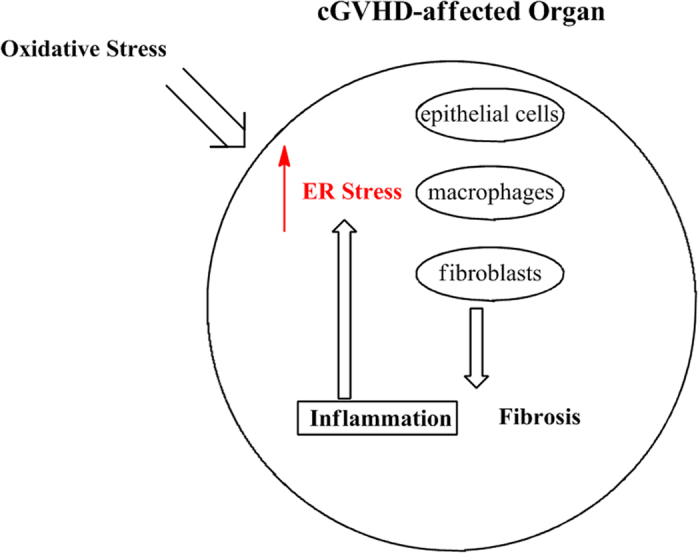
Conceivable association between cGVHD and ER stress. cGVHD-affected organs are conceivably subjected to oxidative stress. Accordingly, ER stress in epithelial cells, fibroblasts and macrophages can be augmented. As a result, (1) inflammation-associated molecules such as NF-κB and TXNIP are expressed and/or activated in an out-of-control manner, (2) fibroblasts are driven to be dysfunctional and whereby produce abnormal collagen bundles and (3) macrophages are caused to differentiate into an M2 phenotype, which can be associated with aberrant fibrosis. In addition, ER stress is thought to be increased by inflammation. Hence, a vicious cycle between ER stress and inflammation can be formed in organs disordered by cGVHD. Thus, reduction of cGVHD-caused ER stress can also break the negative cycle. Altogether, the ER stress reducer PBA can be an effective drug to treat cGVHD.

cGVHD is a major complication following HSCT and can be deadly for some patients. Although cGVHD has been studied intensively over the last decades, sluggish progress in developing efficacious treatment of cGVHD has been made. Patients with cGVHD have been treated with immunosuppressant such as cyclosporine in many cases, however the desired results have been elusive. Hence, clinical settings are in desperate need of effective methods to cure cGVHD. A survey of literature suggests (1) that cellular senescence in donor immune cells and recipient cells detrimentally contributes to the pathogenic process of cGVHD^14^ and (2) that ER stress serves a detrimental role in the development of chronic inflammation and age-associated diseases^29^,^30^. These reports have led us to a new hypothesis (1) that cGVHD-affected organs show the elevated level of ER stress and (2) that alleviation of ER stress can be a robust strategy to ease severe symptoms caused by systemic cGVHD (Fig. 7).

In this proof-of-concept research, cGVHD was experimentally induced in mice. We investigated the pathogenesis of chronic GVHD in the human lacrimal glands from 2001 to 2009^32^,^38^,^39^,^40^,^41^. Our investigation demonstrated that the pathogenesis of lacrimal gland cGVHD in this murine model was reminiscent of that in patients with chronic GVHD^1^,^14^,^31^,^42^. Excessive inflammation and fibrosis observed in this mouse model greatly resemble those seen in human samples. Furthermore, our previous reports showed that systemic inflammation and fibrosis were observed in this mouse model^1^,^31^. Hence, we chose this animal model to tackle systemic cGVHD.

Toward the ultimate goal, we initially elucidated (1) the elevation of ER stress and (2) the increased activity and/or expression of its corresponding proinflammatory molecules such as NF-κB and TXNIP in cGVHD-affected organs. Drobyski and co-workers have demonstrated that NF-κB detrimentally correlates with the pathogenic process of aGVHD and that inhibition of NF-κB can alleviate aGVHD^5^. Our study suggests that NF-κB is activated in cGVHD-affected organs and that it results partly from unresolved ER stress caused by cGVHD.

With respect to the involvement of TXNIP in the pathogenesis of cGVHD, there is no literature precedent which mentioned an association between TXNIP and cGVHD. Our survey of previous articles has revealed (1) that TXNIP is generated in the downstream of the PERK and IRE1α pathways, (2) that it serves a pivotal role in a crossroad where cells subjected to ER stress determine whether to restore homeostasis or undergo apoptosis and (3) that it induces extensive inflammation by activating the inflammasome nod-like receptor family, pyrin domain containing 3 (NLRP3)^22^,^23^,^24^. Thus, our investigation indicates that the ER stress-elicited expression of TXNIP can correlate with disabling symptoms of cGVHD.

We obtained these scientifically and medically important outcomes, and our subsequent endeavors were to develop effective therapeutics to treat cGVHD through mitigation of ER stress. We initially attempted to reduce cGVHD-induced ER stress utilizing the ER stress mitigator rapamycin^4^,^43^,^44^,^45^ (Supplementary Figure 1B) and found that it could ease ER stress and be efficacious for systemic cGVHD (data not shown). However, it also acts as an immunosuppressive drug and has been reported to induce extensive side-effects such as viral and fungal infections in clinical settings^46^,^47^. These reports prompted us to circumvent the setbacks and search for a safer compound to mitigate ER stress, and we chose 4-phenylbutyric acid (PBA), which is a commercially available aromatic fatty acid and can be used as an affordable starting material in synthetic organic chemistry^48^,^49^. In medical practice, PBA has been proven to be sufficiently safe to treat urea cycle disorders^27^. Literature precedent suggests that PBA can attenuate ER stress, since it serves as a chemical chaperone and assists the ER in dealing with unfolded/misfolded proteins^50^. As demonstrated by previous reports, PBA can be effective for type 2 diabetes and obesity-elicited chronic inflammation in adipose tissues by lowering ER stress^29^,^30^. Taken together, it was envisioned that PBA could be a safe and powerful tool to reduce cGVHD-caused ER stress.

A series of our experiments demonstrated that cGVHD-caused ER stress in murine organs could be mitigated by PBA and that accordingly the ER stress-associated proinflammatory molecules NF-κB and TXNIP were greatly subdued.

With these encouraging results, we subsequently investigate the state of cGVHD-vulnerable organs. Histological, immunohistochemical and electron microscopic analysis of these tissues was conducted. The HE pictures and immunofluorescence images of the cGVHD target organs demonstrated that the PBA-medicated tissues were less inflamed than the vehicle-medicated ones. Our survey of previous reports shows (1) that PBA can alleviate inflammation by reducing ER stress in endothelial cells and (2) that when ER stress is elevated, mitochondria are damaged^51^,^52^,^53^. As demonstrated by electron micrographs of the lacrimal glands, when mice were treated with the solvent-vehicle, mitochondria in the blood vessels were impaired. In contrast, this phenomenon was not observed in the lacrimal glands collected from the PBA-medicated mice. Thus, these findings indicate that PBA can protect blood vessels from cGVHD-caused ER stress and thereby suppress immune cell migration to tissues.

Mallory staining and immunoblot analysis of the fibrosis marker CTGF demonstrate that systemic fibrosis was subdued by PBA. Our literature search has revealed that epithelial to mesenchymal transition (EMT) correlates with fibrosis observed in human ocular cGVHD and that TXNIP is linked to EMT^54^,^55^. Hence, PBA is conceivably able to attenuate cGVHD-triggered systemic fibrosis, partly because it precludes EMT by lowering the expression of TXNIP. Furthermore, as indicated by ELISA, sera collected from the PBA-dosed mice displayed the lower levels of the inflammatory molecules MCP-1, TNF-α and IFN-γ in comparison to those obtained from their vehicle-dosed equivalents. CTGF is known to serve as an indicator for fibrosis^56^, and our immunoblot analysis of the cGVHD-susceptible organs indicates that PBA can prevent the cGVHD-elicited overexpression of CTGF. These results have underpinned our claim that reduction of ER stress can attenuate disabling symptoms caused by systemic cGVHD, and have encouraged us to conduct more painstaking investigation into the correlation between ER stress and the pathogenic process of cGVHD. As mentioned above, cGVHD causes extensive fibrosis in various organs. Although there is sparse information about the mechanisms of cGVHD-induced fibrosis, a previous report demonstrates that the murine lacrimal glands impaired by cGVHD have the increased number of activated fibroblasts and that they are associated with the progression of fibrosis^32^. Hence, we focused on fibroblasts in the lacrimal glands and scrutinized a correlation between cGVHD-induced ER stress and disordered fibroblasts. Our data suggest (1) that cGVHD-caused ER stress activates fibroblasts in a detrimental way and thereby triggers extensive fibrosis and (2) that PBA can prevent fibroblasts from dysfunctioning through alleviation of ER stress.

Once we gained novel insights into how ER stress affected the function of fibroblasts, our focus was turned to macrophages. A previous study demonstrates that senescent macrophages serve a causative role in the development of ocular cGVHD^14^. Other workers have shown (1) that ER stress promotes the polarization into M2 macrophages and (2) that M2 macrophages are implicated in fibrosis-related diseases^57^,^58^,^59^. These reports captured our interest and motivated us to scrutinize the relationship between cGVHD-induced ER stress and M2 macrophage differentiation. Judging from immunofluorescence images, the cGVHD-impaired murine lacrimal glands had macrophages expressing the ER stress marker CHOP, whereas it was not the case with the PBA-medicated murine lacrimal glands. These observations indicate that macrophages subjected to ER stress are linked to the progression of cGVHD. Furthermore, splenic macrophages from PBA- and vehicle-medicated mice were induced and subsequently analysed utilizing sophisticated methods. Both immunohistochemistry and immunoblot analysis demonstrated that the PBA-treated splenic macrophages showed the lower protein levels of ER stress markers compared to their vehicle-treated counterparts. Quantitative mRNA analysis suggests (1) that the mRNA levels of the M2 markers TGF-β and IL-10 are enhanced in the splenic macrophages treated with the solvent-vehicle and (2) that the gene expression of the M1 markers IL-1β and IL-6 is increased in those treated with PBA. Hence, it is conceivable (1) that cGVHD-induced ER stress promotes macrophages to differentiate into an alternatively activated phenotype and (2) that PBA can mitigate cGVHD-induced fibrosis by reducing ER stress in macrophages and transforming them into a classically activated phenotype.

Collectively, considering the safety and affordability of the ER stress reducer PBA, the reduction of cGVHD-caused ER stress can be an innovative and robust strategy for the treatment of systemic cGVHD and open up new prospects to cure other immune-mediated diseases associated with ER stress.

## Methods

Eight-week-old B10.D2 and BALB/c mice were purchased from Sankyo Laboratory, Inc. (Tokyo, Japan). All the scientific experiments on mice were performed according to the Animal Welfare Act at Keio University School of Medicine. Our protocols for experiments on animals were approved by the animal care and use committee at Keio University (Approval Number: 09152). We also complied with the ARRIVE guidelines^60^.

### Bone marrow transplantation

Bone marrow transplantation (BMT) was conducted to furnish a murine model of cGVHD^42^. In the case where the donors were B10.D2 mice and the recipients were BALB/c mice, it was allogeneic BMT to produce a murine model of cGVHD. In contrast, BMT from BALB/c to BALB/c mice was syngeneic BMT, and therefore cGVHD did not occur in the transplant recipients. The recipient mice without cGVHD served as syngeneic control subjects. The recipients were irradiated with 700cGy prior to the BMT, and the lethal irradiation was preformed using a Gammacel 137 Cs source (Hitachi Medico, Ltd, Tokyo, Japan). A suspension containing 1 × 10^6^ bone marrow cells and 2 × 10^6^ spleen cells from the donors was administered to each of the recipient mice via tail vein. The donor cells were suspended in RPMI 1640 (Life Technologies Japan Ltd, Tokyo, Japan).

### Treatment of allogeneic BMT recipient mice with PBA

We performed BMT as described in the Methods section, and the allogeneic BMT recipient mice were divided into 2 groups. One group was treated with PBA (10 mg/kg) (Aldrich, St. Louis, MO), and the other was given the solvent-vehicle PBS (pH 7.4) by intraperitoneal injection. We administered PBA or the solvent-vehicle to the allogeneic BMT recipients once per day from Day 10 to Day 27 after BMT. They were sacrificed Day 28 after BMT. In this study, the following cGVHD-susceptible organs were analyzed: extra-orbital lacrimal glands, the proximal part of small intestine, dorsum skin, liver, salivary glands, lung, large intestine and eyes.

### Culture of fibroblasts from murine lacrimal glands

Fibroblasts derived from the murine lacrimal glands were cultured using the well-established method reported by Yaguchi *et al*.^35^. The murine lacrimal glands were collected and subsequently cut into small pieces. The tissue fragments were incubated at 37 °C in DMEM (Life Technologies Japan Ltd, Tokyo, Japan) containing 5% of fetal bovine serum (FBS) (Sigma, St. Louis, MO) and 5% of antibiotics. The antibiotics were a 1:1 aqueous mixture of streptomycin sulfate (Meiji Seika Pharma Co., Ltd, Tokyo, Japan) and benzylpenicillin potassium (Meiji Seika). Fibroblasts started growing from the minced pieces after 3–4 days. The fibroblasts were cultured in DMEM with 5% of FBS and 5% of antibiotics, and used for experiments after 3–5 passages. Trypsin (Becton Dickinson, Franklin Lakes, NJ) was used to detach the fibroblasts from the culture dishes.

### Culture of splenic macrophages

We cultured murine splenic macrophages according to the method of Alatery and Besta with slight modifications^36^. Splenocytes suspended in RPMI with 5% of FBS were seeded in culture dishes and left at 37 °C overnight. The floating cells were then removed. The desired cells attached to the dishes were cultured in RPMI containing recombinant murine M-CSF (5 ng/mL) (Peprotech, Rocky Hill, NJ), 5% of FBS and 5% of antibiotics for 7 days. The induced macrophages were subsequently detached from the culture dishes utilizing accutase (Thermo Fisher Scientific, Waltham, MA) and used for experiments.

### Immunostaining of cultured macrophages

The induced macrophages were placed in 8-well chamber slides (Fibronectin Culture Slide; Corning Inc., Corning, NY) and fixed with 10% neutral buffered formlain. They were then blocked with methanol containing 0.3% hydrogen peroxide at RT for 30 min and incubated with the primary antibodies CD68 (AbD Serotec) and CHOP (Santa Cruz) at 4 °C overnight. The sections were subsequently treated with goat anti-mouse IgG (H + L) secondary antibody, Alexa Fluor 568 conjugate (Molecular Probes, Eugene, OR) and 4′,6-diamidino-2-phenylindole (DAPI) (Life Technologies) at RT for 45 minutes and mounted with an anti-fading mounting medium (Dako). Fluorescence images were taken with an LSM confocal microscope (Carl Zeiss).

### Quantitative polymerase chain reaction

Total RNA was extracted from extra-orbital lacrimal glands, the proximal part of small intestine, dorsum skin, liver, and cultured fibroblasts and macrophages using an miRNeasy mini kit (Qiagen, Valencia, CA), and the corresponding complementary DNA was synthesized utilizing a Rever Tra Ace qPCR RT Kit (Toyobo Co. Ltd. Osaka, Japan). The primers required to analyze the mRNA expression of the following genes by the use of TaqMan real-time polymerase chain reaction (PCR) were purchased from Applied Biosystems (Applied Systems Inc, Streetsville, Ontario, Canada): the housekeeping gene glyceraldehyde 3-phosphate dehydrogenase (GAPDH), glucose regulated protein 78 (GRP78), Interleukin-1β (IL-1β), IL-6, IL-10, macrophage chemoattractant protein-1 (MCP-1) and transcription growth factor-β (TGF-β). Quantitative real-time PCR was performed using the Step One Plus system (Applied Biosystems). The 2^−ΔΔCT^ method was used to analyze the data obtained, and GAPDH was used as an internal standard to measure the expression of mRNA.

### Statistical analysis

Statistical significance was determined by the use of unpaired Mann–Whitney U test. Differences are considered significant in the case of P < 0.05. The acquired data are presented as means ± SD.

## Additional Information

**How to cite this article**: Mukai, S. *et al*. Novel Treatment of Chronic Graft-Versus-Host Disease in Mice Using the ER Stress Reducer 4-Phenylbutyric Acid. *Sci. Rep.* **7**, 41939; doi: 10.1038/srep41939 (2017).

**Publisher's note:** Springer Nature remains neutral with regard to jurisdictional claims in published maps and institutional affiliations.

## Supplementary Material

Supplementary Information
