# Supplementary material for: Novel Treatment of Chronic Graft-Versus-Host Disease in Mice Using the ER Stress Reducer 4-Phenylbutyric Acid
Source: Sci Rep. 2017 Feb 6;7:41939. doi: 10.1038/srep41939 (PMC5292729; doi:10.1038/srep41939)
Supplement: Supplementary Information [file srep41939-s1.pdf]

Novel Treatment of Chronic Graft-Versus-Host Disease in Mice Using the ER Stress Reducer 4-Phenylbutyric Acid

Shin Mukai,[1, 3] Yoko Ogawa,[1] Fumihiko Urano,[2] Chie Kudo-Saito,[3] Yutaka Kawakami,[3] Kazuo Tsubota[1]

[1]Deaprtment of Ophthalmology, Keio University School of Medicine, [2]Department of Medicine, Division of Endocrinology, Metabolism, and Lipid Research, and Department of Pathology and Immunology, Washington University School of Medicine, [3]Institute for Advanced Medical Research, Keio University School of Medicine.

This study was supported by the Japanese Ministry of Education, Science, Sports and Culture, #26462668

Supplementary Methods

Histological analysis and immunohistochemistry

Three or four weeks after BMT, extra-orbital lacrimal glands, the proximal part of small intestine, dorsum skin, liver, salivary glands, lung, large intestine and eyes were collected from the transplant recipients. These samples were subsequently fixed with 10% neutral-buffered formalin and embedded in paraffin. The paraffin blocks were cut into 7μm-thick sections, and then stained with (1) hematoxylin and eosin, (2) Mallory's trichrome[1, 2] and (3) antibodies used in this study. For immunohistochemical assays, paraffin was removed in the first instance, followed by the recovery of the antigens using either of the following 2 antigen retrieval methods. (A) To stain the sections with a CD45 antibody (30-F11, BD Pharmingen, San Jose, CA), they were immersed in the antigen retrieval solution (Target Retrieval Solution; Dako, Glostrup, Denmark) and then boiled with a microwave oven for 10 min. (B) In the case of multiple staining for CD68 (FA-11, AbD Serotec, Kidlington, UK) and CHOP (F-168, Santa Cruz Biotechnology, Santa Cruz, CA), the sections were soaked in the antigen retrieval solution (HistoVT One; Nakalai Tesque, Kyoto, Japan) and subsequently heated at 90 °C for 40 min with a water bath. Next, the sections were blocked with 10% normal goat serum, and the reactions between the antigens in tissue sections and the primary antibodies were conducted at 4°C overnight. The sections were then treated with fluorophore-labelled secondary antibodies at RT for 45 minutes and mounted with an anti-fading mounting medium (Fluorescent Mounting Medium; Dako). Fluorescence images were taken with an LSM confocal microscope (Carl Zeiss, Jena, Germany). As for the counting of $CD45^+$ cells, five areas of each tissue section were randomly photographed under 200X magnification, and the number of $CD45^+$ cells in the individual images was subsequently determined.

The following secondary antibodies were used in this study: goat anti-mouse IgG (H+L) secondary antibody, Alexa Fluor 488 conjugate (Molecular Probes, Eugene, OR) and goat anti-rat IgG (H+L) secondary antibody, Alexa Fluor 568 conjugate (Molecular Probes). With respect to isotype controls, rat IgG2b, κ (eB149/10H5, eBioscience, San Diego, CA), rat IgG2a (54447, R&D Systems, Minneapolis, MN) and rabbit IgG (Cell Signaling Technology, Danvers, MA) were utilized for CD45, CD68 and CHOP, respectively.

Electron microscopy

Transmission electron microscopic analysis was performed according to standard protocols. Tissues were collected from the murine lacrimal glands and small intestine, immediately fixed with 2.5% glutaraldehyde in 0.1 M phosphate buffer (pH 7.4) at 4°C for 4 hours and washed three times with 0.1 M phosphate buffer. The samples were subsequently fixed again with 2% osmium tetroxide, dehydrated in a graded series of ethanol and 100% propylene oxide, and embedded in epoxy resin. One micrometer sections were made from the processed tissues and then stained with methylene blue. The thick sections were observed with a microscope to find parts which were suitable for preparation of ultrathin sections. The obtained sections were placed on mesh grids, stained with uranylacetate and lead citrate, and examined with an electron microscope (1230 EXII; JOEL, Tokyo, Japan). All electron micrographs were acquired with a bio scan camera (Gatan bio scan camera model 792, Tokyo, Japan).

Immunoblotting analysis

The tissues of interest were placed in Eppendorf tubes, and pre-cooled RIPA buffer was added to the tubes. The tissues were then homogenized using an electric homogenizer. After the samples were on ice for 1h, they were centrifuged at 15000 rpm at 4°C for 5 min. The supernatants were subsequently collected in fresh tubes on ice and used as cell lysates. An

equal amount of 5X Laemmli buffer was added to each cell lysate, followed by protein denaturation at 100°C for 5 min. Equal amounts of protein from each sample were loaded into the wells of SDS-PAGE gels and then resolved. The proteins were transferred from the gels to membranes at 15 V for 20 min. The membranes were blocked with 5% skim milk or 5% BSA in 1 x TBST (a mixture of tris-buffered saline and tween 20) at RT for 1h. The membranes were then incubated with primary antibodies at 4°C overnight. The primary antibodies were diluted 1000 times with 5% skim milk or 5% BSA in 1 x TBST. After the primary antibody incubation, the membranes were washed with 1 x TBST (3 x 10 min), subjected to secondary antibody at RT for 1h and then washed with 1 x TBST (3 x 10 min) and 1 x TBS (2 x 10 min). The proteins of interest were visualized using either of the following two methods. (1) Colorimetric detection of the target proteins was conducted using BCIP/NBT substrate (Promega, WI). (2) Signals were developed with an enhanced chemoluminescence (ECL) detection reagent (GE Healthcare, Littlecalfont, UK), and the target proteins were subsequently visualized with a LAS 4000 mini chemiluminescence imaging system (Fujifilm/GE Healthcare). Densitometric analysis of the obtained protein bands was conducted by the use of the image processing software ImageJ. The primary antibodies used in this experiment were as follows: GRP78 (Abcam, Cambridge, UK), phospho-PERK (Thr980, Cell Signaling Technology), PERK (C33E10, Cell Signaling Technology), phosphor-IRE1α (Thermo Fisher Sceientific, Waltham, MA), IRE1α (14C10 Cell Signaling Technology), phosphor-eIF2α (119A11, Cell Signaling Technology), eIF2α (Cell Signaling Technology), CHOP (9C8, Thermo Fisher Scientific), TXNIP (D5F3E, Cell Signaling Technology) NF-κB (Abcam), HSP47 (SPA-470, Stress Gen Biotechnologies Corp, San Diego, CA), CTGF (Abcam), α-SMA (1A4, Abcam), CD68 (FA-11, Abcam), Cytokeratin (C-11, Abcam), CD3 (Abcam), CD19 (Cell Signaling Technology), CD20 (D-10,

Santa Cruz) and β-actin (AC-15, Abcam). With regards to the secondary antibodies, (1) when the protein bands were visualized by developing a color, either an AP-conjugated anti-mouse IgG antibody (Promega), an AP-conjugated anti-rabbit IgG antibody (Promega) or AP-conjugated anti-rat IgG antibody (Promega) was used, and (2) either an HRP-conjugated anti-mouse antibody (Thermo Fisher Scientific) or an HRP-conjugated anti-rabbit antibody (Thermo Fisher Scientific) was required to detect the target proteins by ECL.

Enzyme linked immunosorbent assay (ELISA)

Blood was collected from PBA- and vehicle-medicated mice and subsequently centrifuged at 4000 rpm for 10 min. The levels of MCP-1, tumor necrosis factor-α (TNF-α), and interferon-γ (IFN-γ) in the obtained sera were measured utilizing ELISA sets (Becton Dickinson). These assays were conducted according to the protocols provided by the manufacturer Becton Dickinson.

Supplementary figures

Supplementary Figure 1

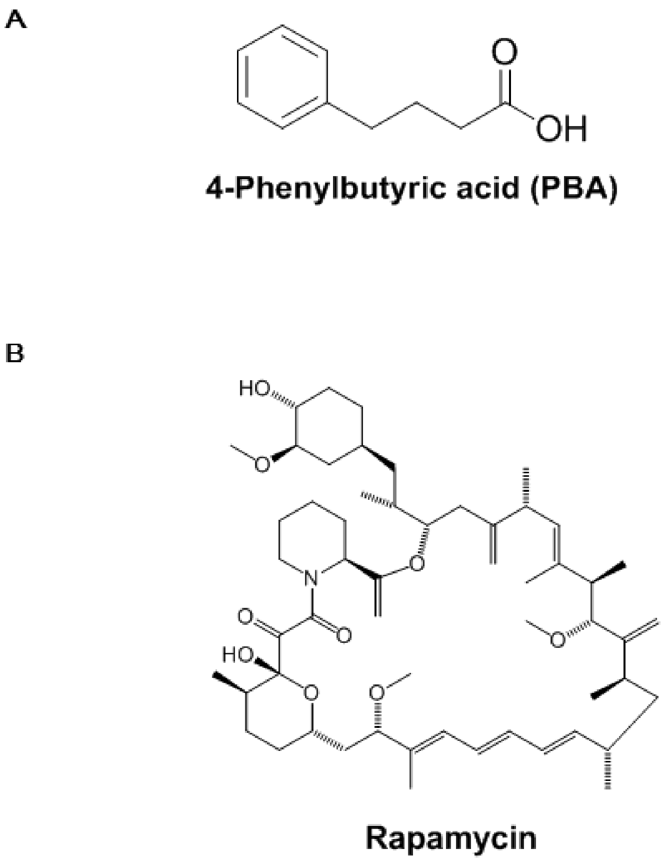

Supplementary Figure 1. Structures of the ER stress reducers (A) 4-phenylbutyric acid (PBA) and (B) rapamycin

Supplementary Figure 2

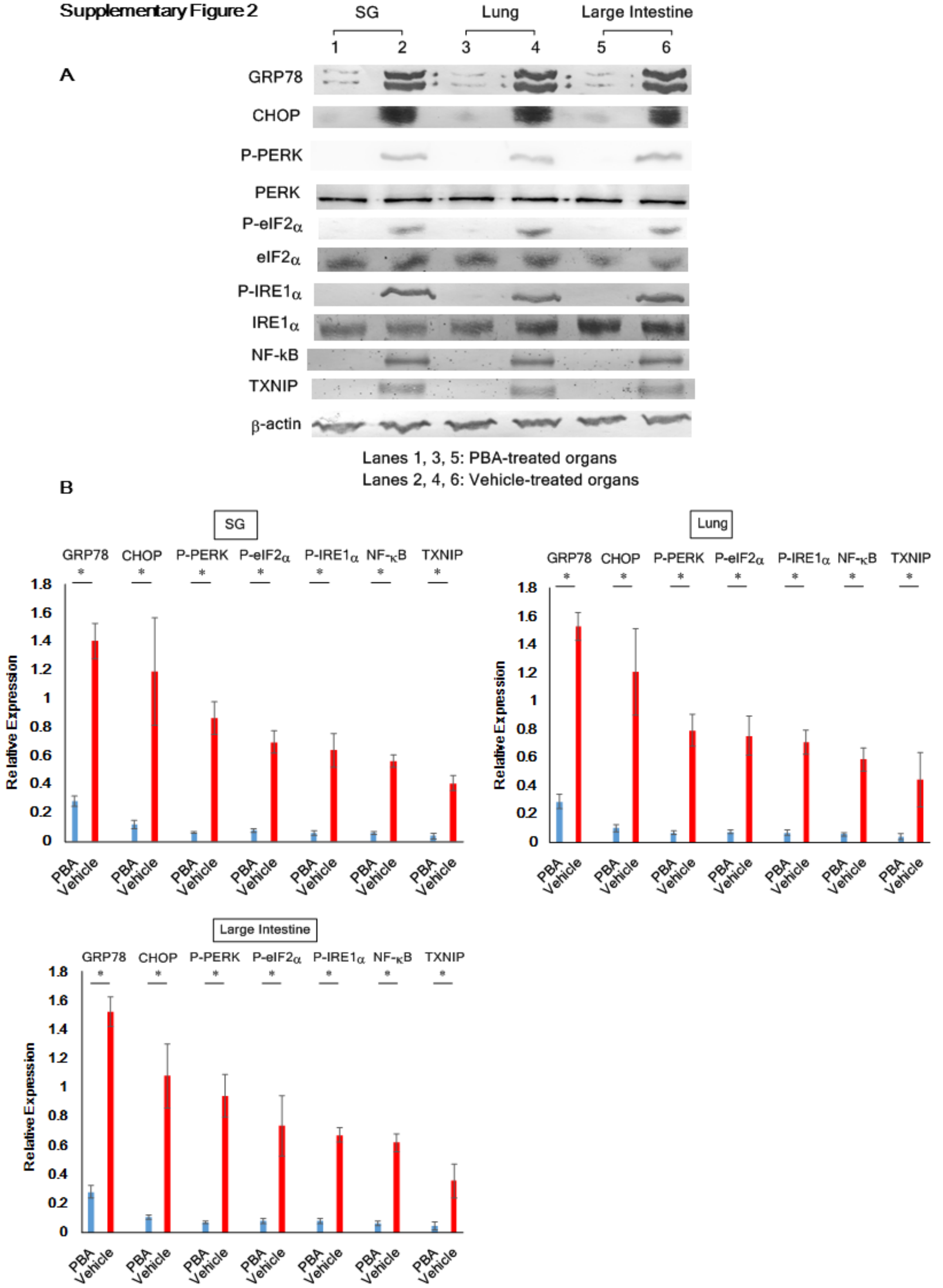

Supplementary Figure 2. Suppression of cGVHD-caused ER stress by PBA. (A) Immunoblot assays of ER stress markers and the associated inflammatory molecules in cGVHD target organs. (Lanes 1, 3, 5: PBA-medicated organs. Lanes 2, 4, 6: Vehicle-medicated organs) Cropped blots are displayed. (B) The target proteins in each organ were subsequently quantified by densitometry. PBA-treated organs (blue) and vehicle-treated organs (red). Data from one of two similar experiments are shown. The data are presented as means, ± SD, PBA: n=4, Vehicle: n=4 *P<0.05.

Supplementary Figure 3

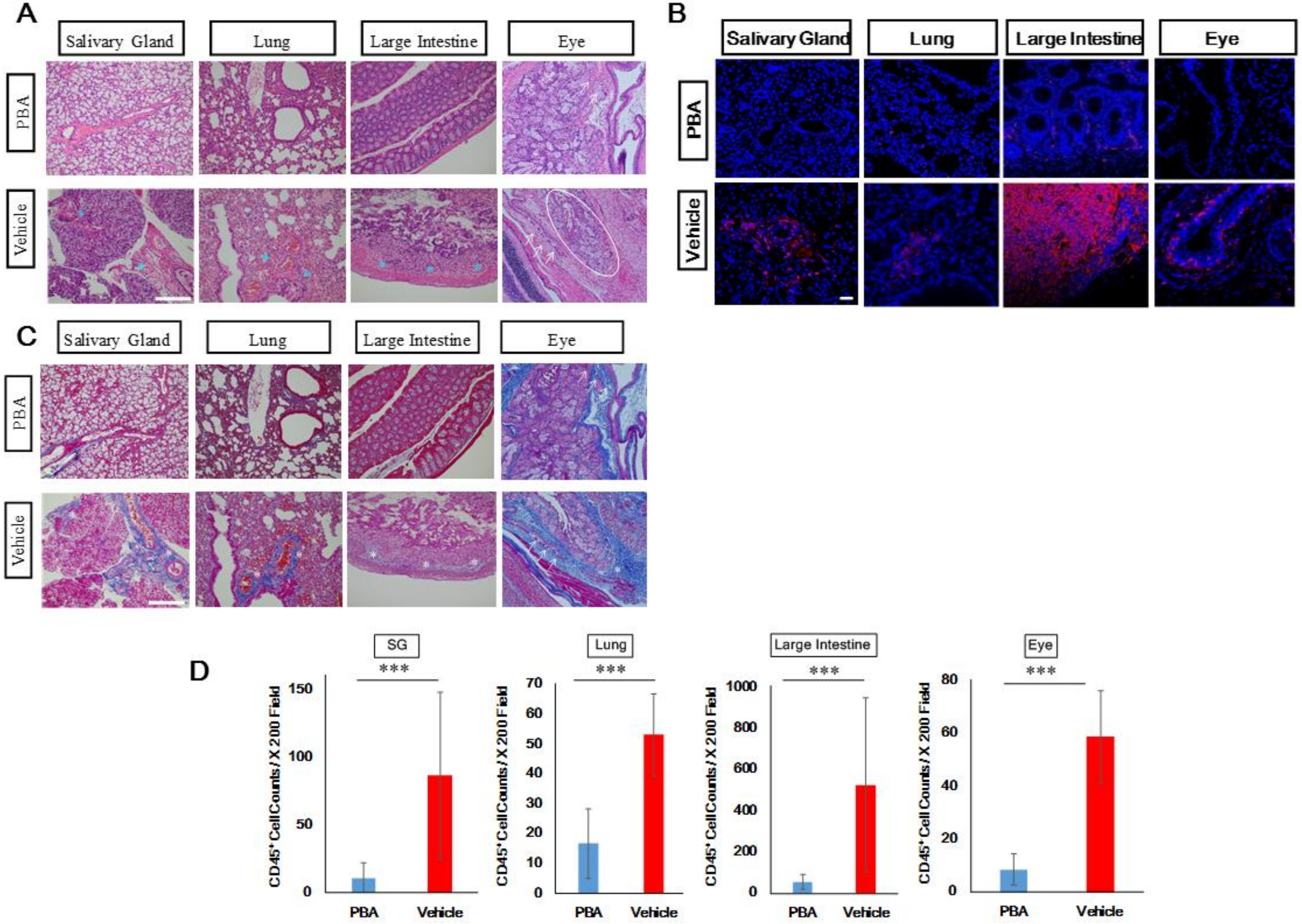

Supplementary Figure 3. Mitigation of cGVHD-elicited systemic inflammation and fibrosis using the ER stress mitigator PBA. (A) HE pictures of the PBA-treated organs and those treated with the solvent-vehicle. The images were taken at 200x magnification, and the scale bar is 200 μm. Severely inflamed portions are shown with asterisks. In the pictures of the vehicle-medicated eye, an ellipse is placed where its meibomian glands were decreased and shrunk, and conjunctival epithelia are indicated with arrows. (B) Immunostaining for the generic leukocyte marker CD45 in the PBA-medicated tissues and their vehicle-medicated equivalents. $CD45^+$ cells and cell nuclei were stained red and blue, respectively. The images were taken at 200x magnification, and the scale bar is 20 μm. (C) Mallory's staining for the PBA-injected organs and their vehicle-injected counterparts. The photographs were taken at 200x magnification, and the scale bar is 200 μm. Aberrantly fibrotic areas are shown with white asterisks. (D) The density of $CD45^+$ cells in the PBA-treated and their vehicle-treated counterparts. PBA-treated organs (blue) and vehicle-treated organs (red). Data from one of two similar experiments are shown. The data are presented as means, ± SD, PBA n=3, Vehicle: n=3, *P<0.05, **P<0.01, ***P<0.001.

Supplementary Figure 4

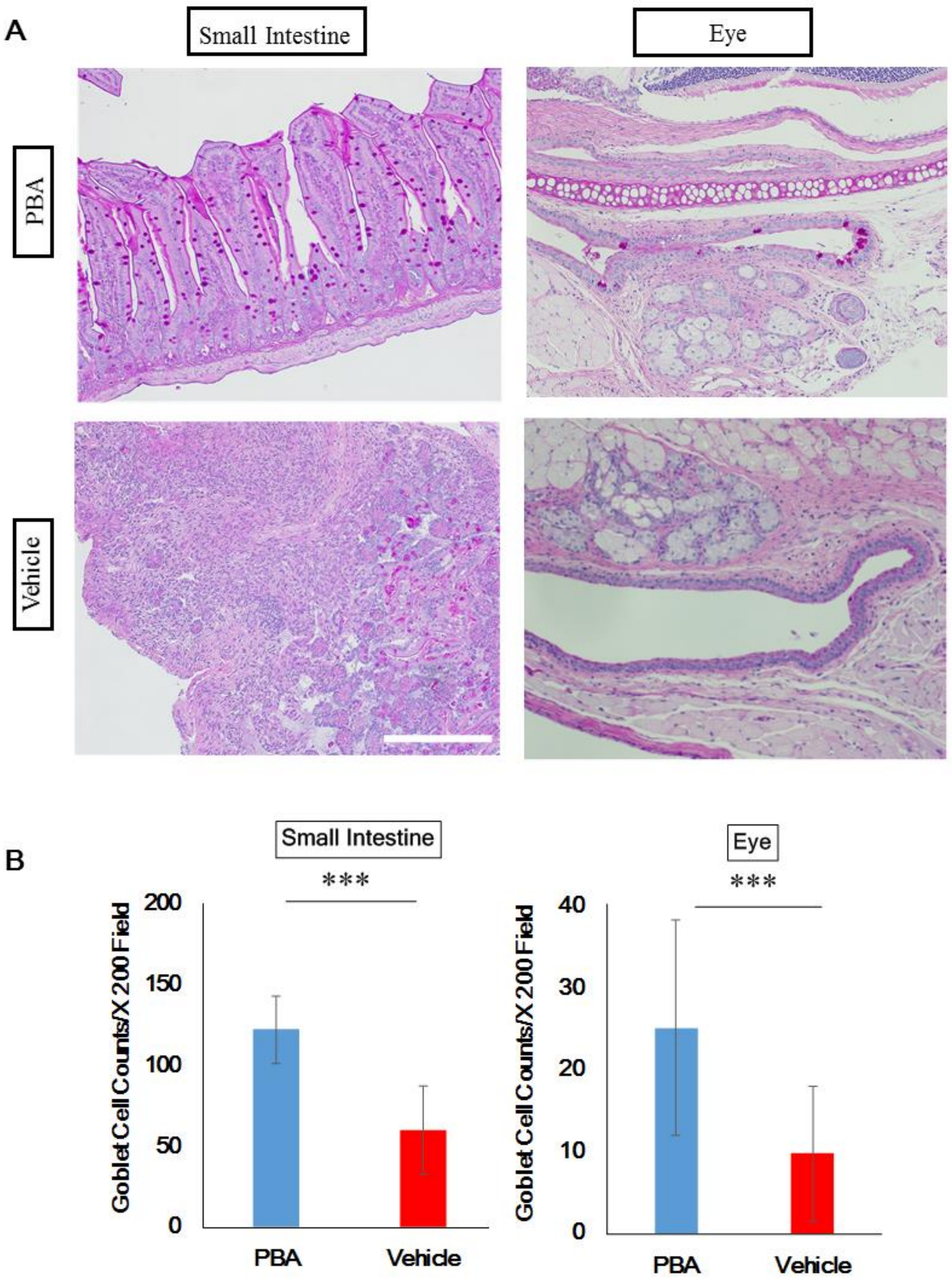

Supplementary Figure 4. Protection of intestinal and conjunctival goblet cells by the ER stress attenuator PBA. (A) PAS staining for the PBA-medicated small intestine and eyes and their vehicle-medicated counterparts. Purple dots in the pictures are goblet cells. The images were taken at 200x magnification, and the scale bar is 200 μm. (B) The density of goblet cells in the PBA-treated small intestine and eyes (blue), and their vehicle-treated counterparts (red). The values are presented as means, ± SD, PBA: n=3, Vehicle: n=3 (small intestine), PBA: n=5, Vehicle: n=5 (eye) ***P<0.001.

Supplementary Figure 5

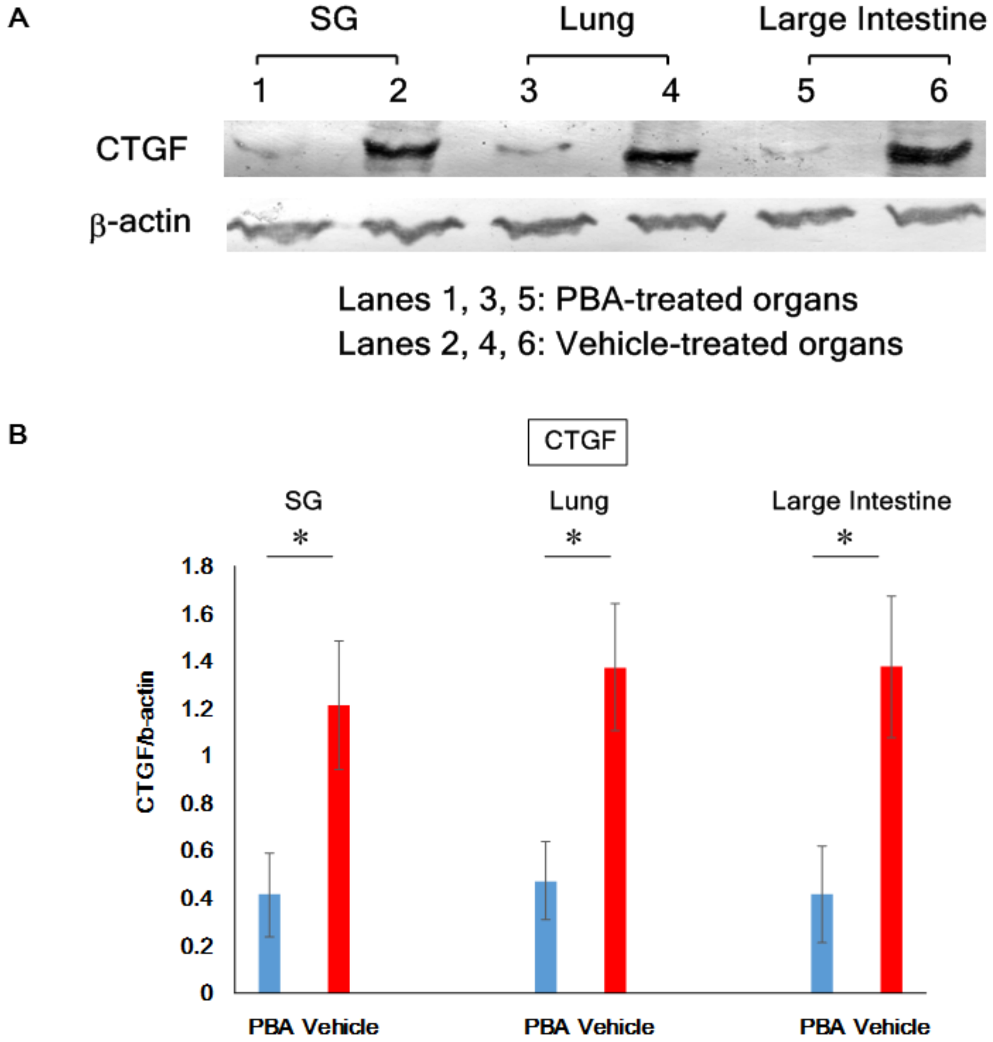

Supplementary Figure 5. Reduction of fibrotic indicators utilizing the ER stress alleviator PBA (A) Immunoblot analysis of the fibrotic marker CTGF. (Lanes 1, 3, 5: PBA-treated organs, Lanes 2, 4, 6: vehicle-treated organs) Cropped blots are displayed. (B) The subsequent densitometric analysis of CTGF in each organ. PBA-treated organs (blue) and vehicle-treated organs (red). Data from one of two similar experiments are shown. The data are presented as means, ± SD, PBA: n=4, Vehicle: n=4 *P<0.05.

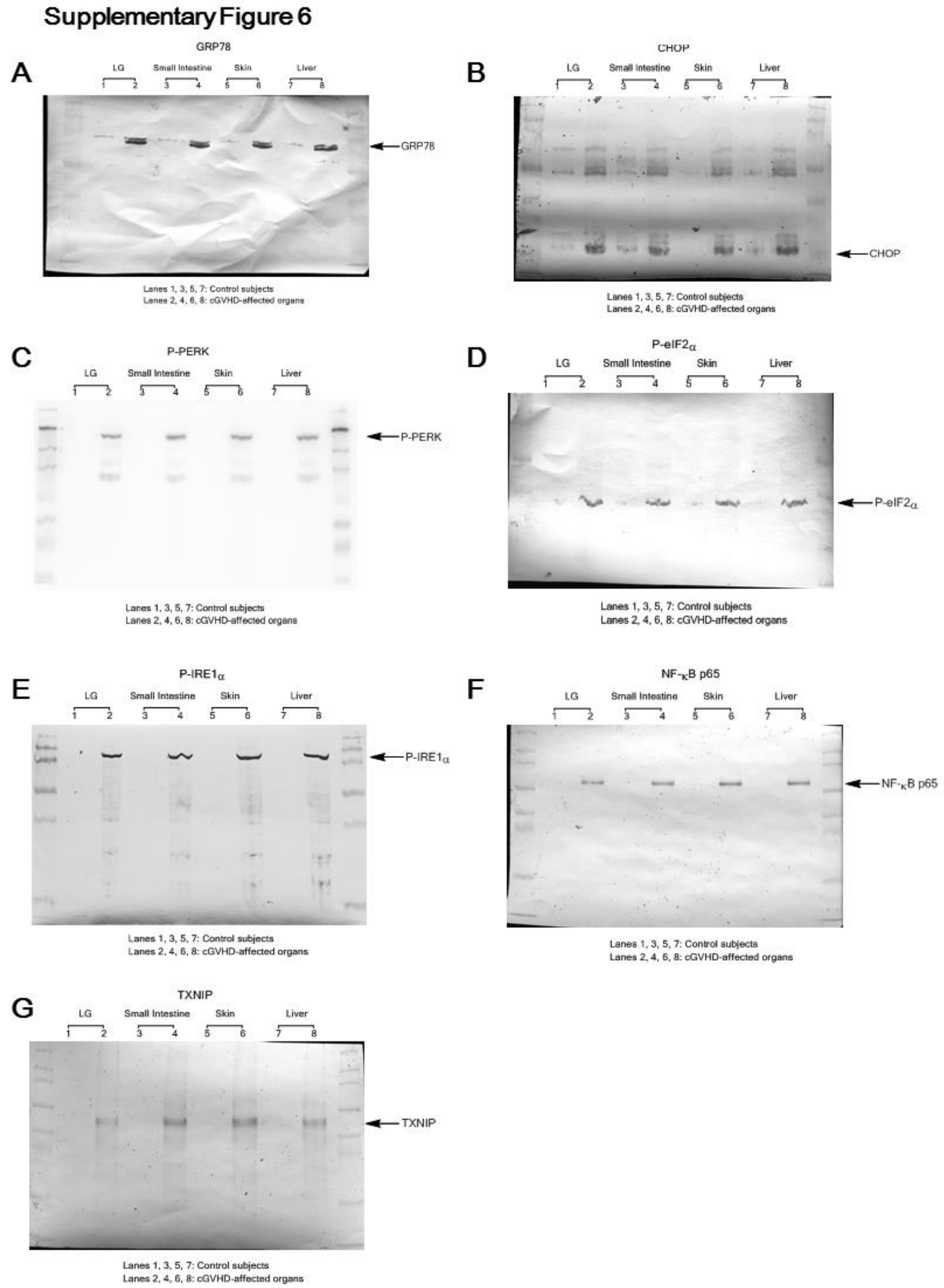

Supplementary Figure 6. Full-length gels from immunoblot assays for the ER stress markers and inflammation-associated molecules shown in Figure 1B. Lanes 1, 3, 5, 7: Syngeneic control subjects, Lanes 2, 4, 6, 8: cGVHD-impaired organs. (A) GRP78, (B) CHOP, (C) P-PERK, (D) P-eIF2α, (E) P-IRE1α, (F) NF-κB p65 and (G) TXNIP

Supplementary Figure 7

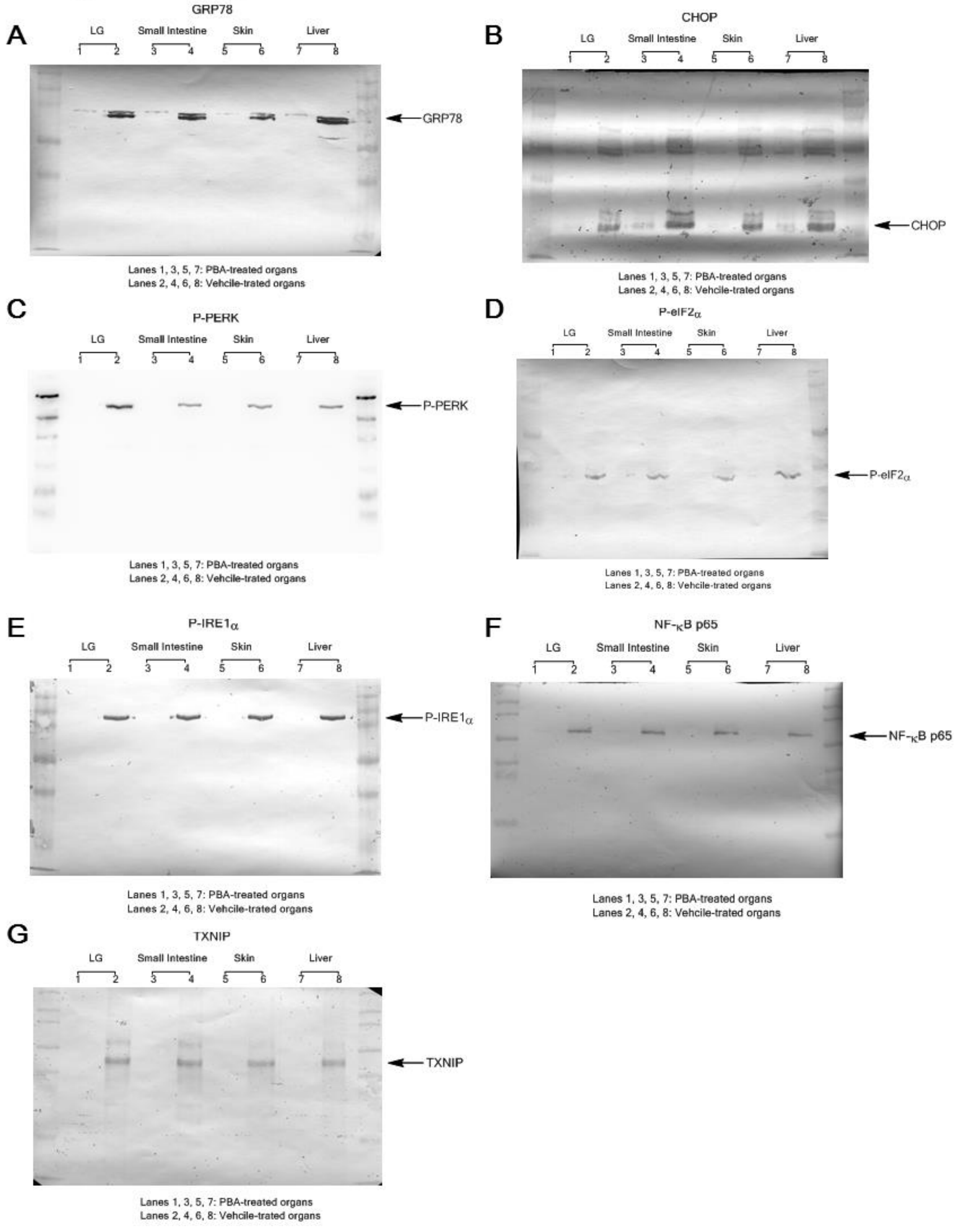

Supplementary Figure 7. Full-length gels from immunoblot assays for the ER stress markers and inflammation-associated molecules shown in Figure 2A. Lanes 1, 3, 5, 7: PBA-medicated organs. Lanes 2, 4, 6, 8: Vehicle-medicated organs. (A) GRP78, (B) CHOP, (C) P-PERK, (D) P-eIF2α, (E) P-IRE1α, (F) NF-κB p65 and (G) TXNIP

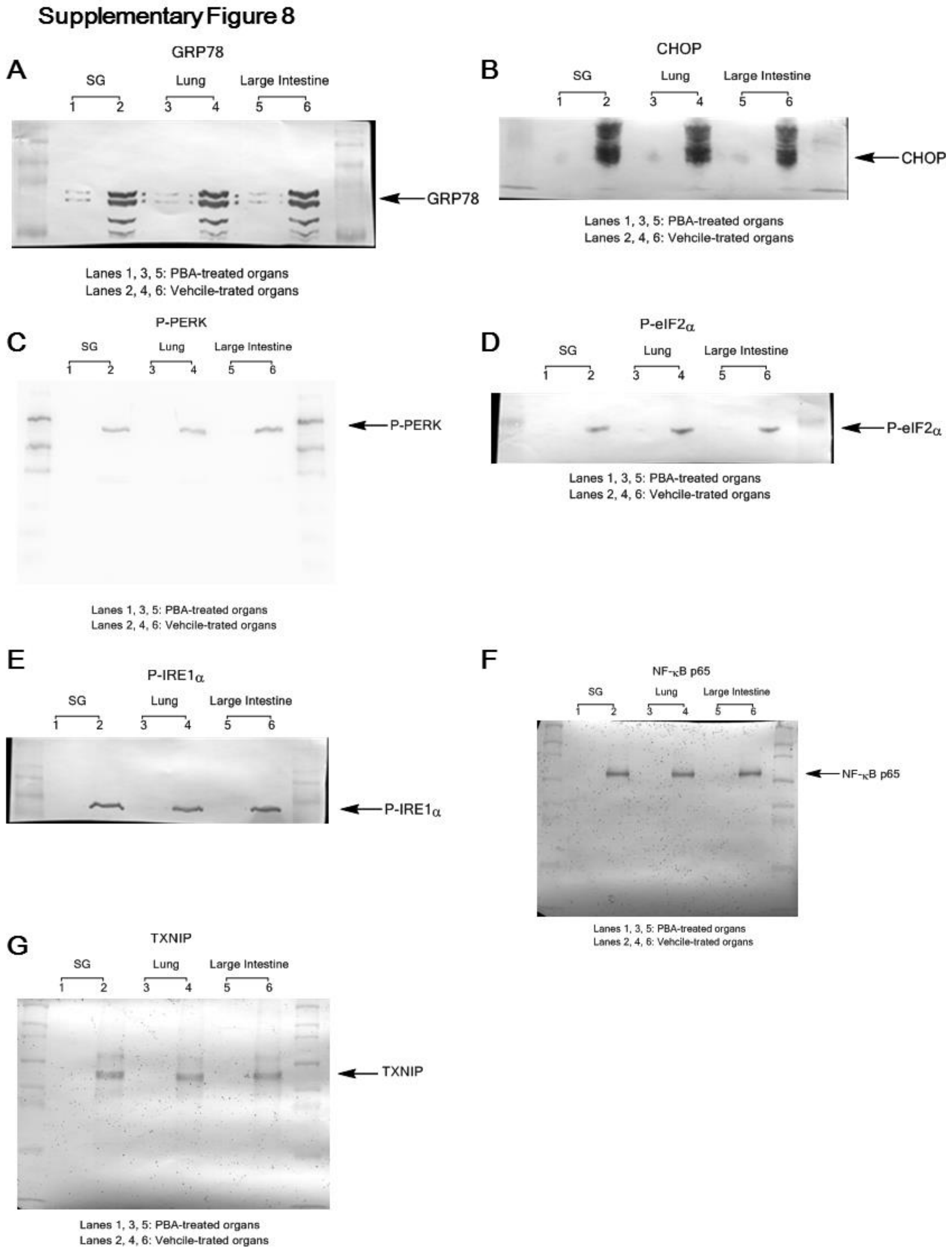

Supplementary Figure 8. Full-length gels from immunoblot assays for the ER stress markers and inflammation-associated molecules shown in Supplementary Figure 2A. Lanes 1, 3, 5: PBA-medicated organs. Lanes 2, 4, 6: Vehicle-medicated organs. (A) GRP78, (B) CHOP, (C) P-PERK, (D) P-eIF2α, (E) P-IRE1α, (F) NF-κB p65 and (G) TXNIP

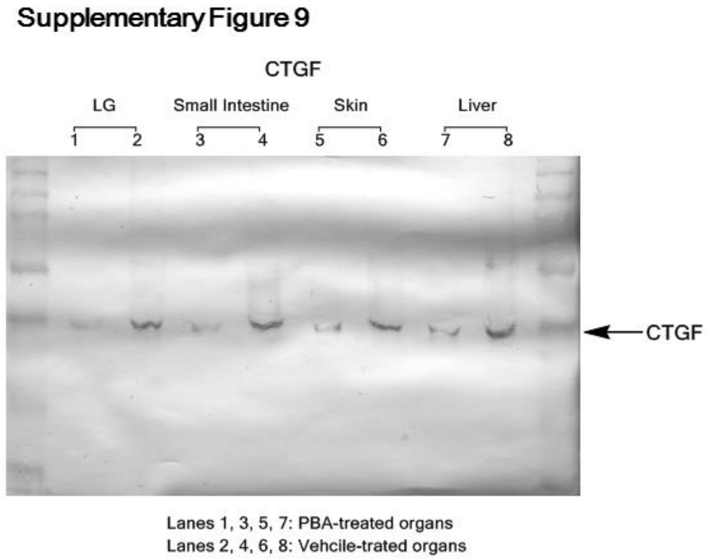

Supplementary Figure 9. Full-length gel from immunoblot analysis of the fibrotic marker CTGF shown in Figure 4B. Lanes 1, 3, 5, 7: PBA-medicated organs. Lanes 2, 4, 6, 8: Vehicle-medicated organs.

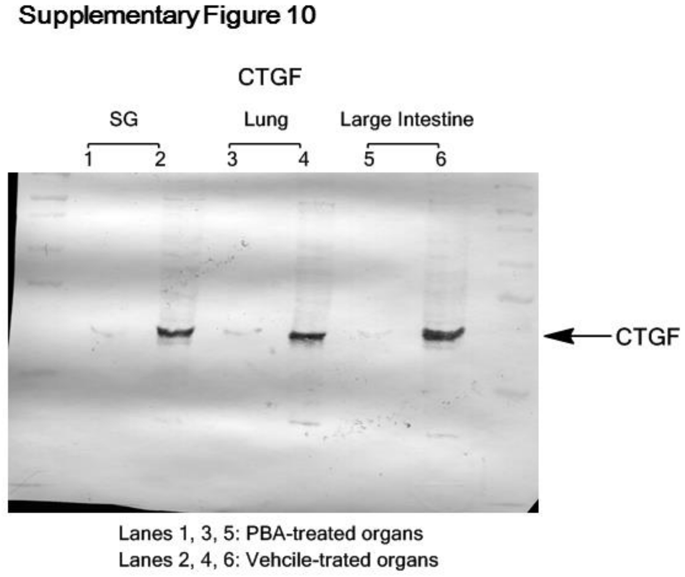

Supplementary Figure 10. Full-length gel from immunoblot analysis of the fibrotic marker CTGF shown in Supplementary Figure 5A. Lanes 1, 3, 5: PBA-medicated organs. Lanes 2, 4, 6: Vehicle-medicated organs.

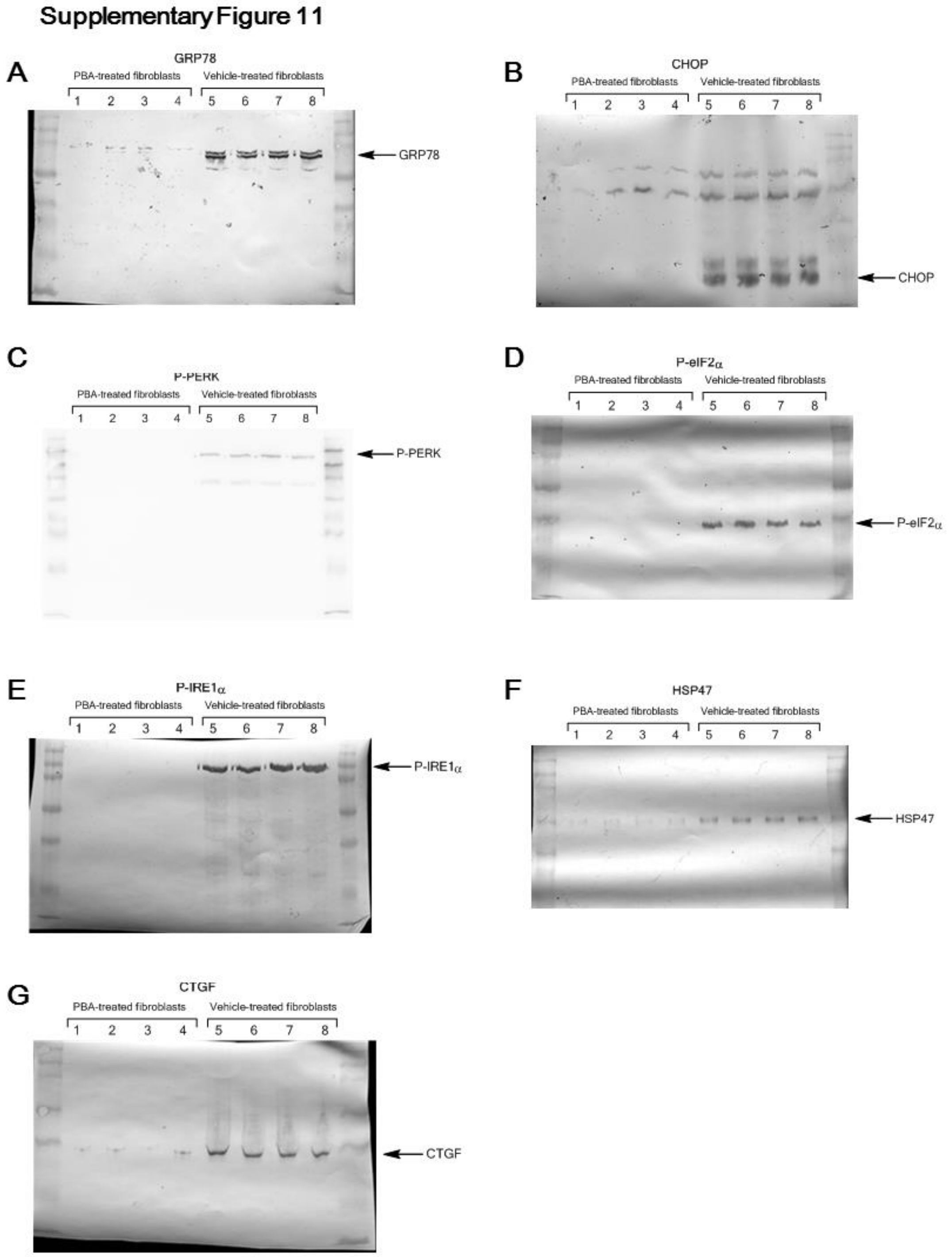

Supplementary Figure 11. Full-length gels from immunoblot assays for the ER stress, activation and fibrotic markers shown in Figure 5A. (Lanes 1, 2, 3, 4: Fibroblasts from the PBA-treated lacrimal glands, Lanes 5, 6, 7, 8: Fibroblasts from the vehicle-treated lacrimal glands). (A) GRP78, (B) CHOP, (C) P-PERK, (D) P-eIF2α, (E) P-IRE1α, (F) HSP47 and (G) CTGF

Supplementary Figure 12

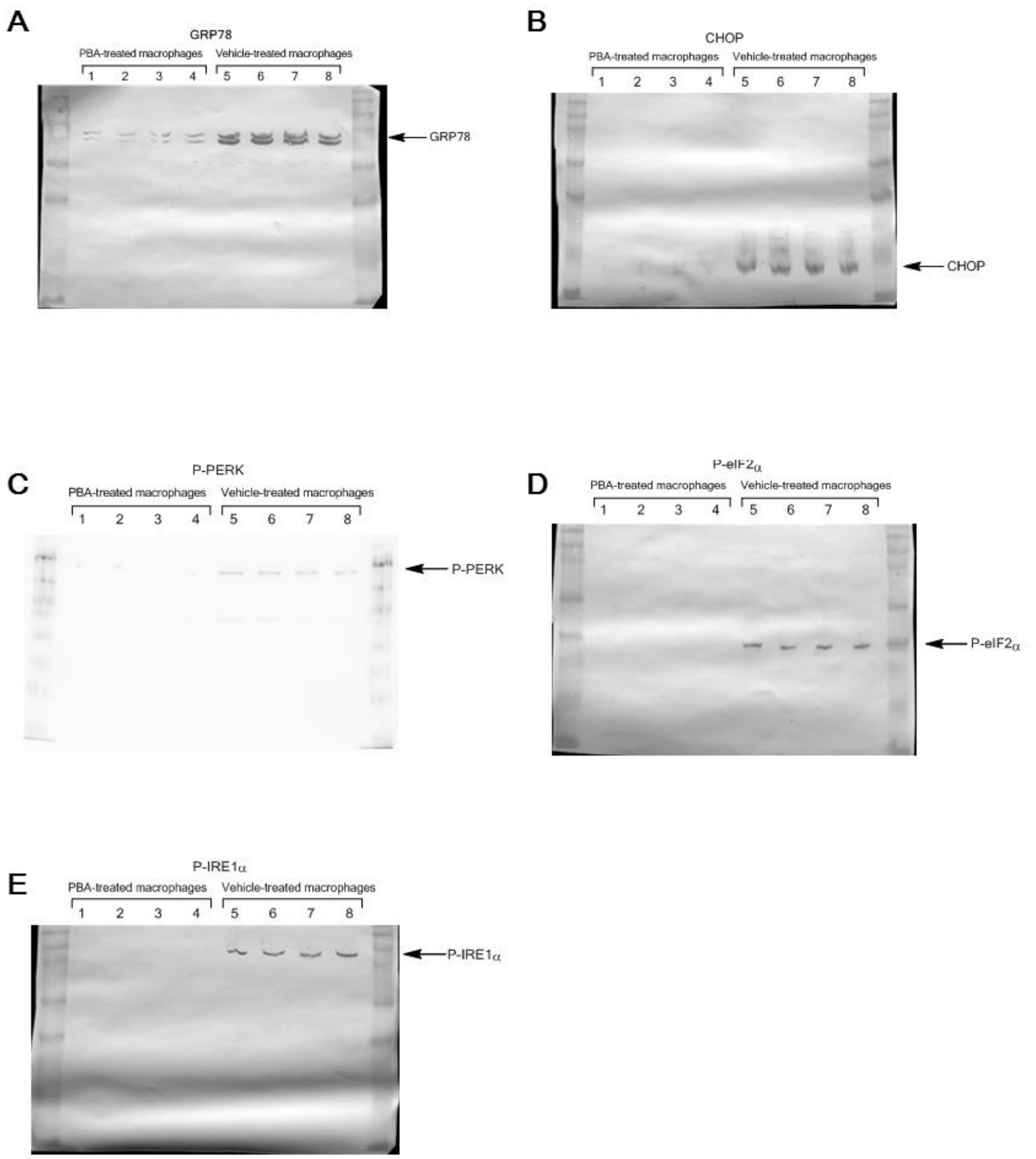

Supplementary Figure 12. Full-length gels from immunoblot assays for the ER stress markers shown in Figure 6C. Lanes 1, 2, 3, 4: Splenic macrophages from PBA-dosed mice, Lanes 5, 6, 7, 8: Splenic macrophages from vehicle-dosed mice). (A) GRP78, (B) CHOP, (C) P-PERK, (D) P-eIF2α, (E) P-IRE1α

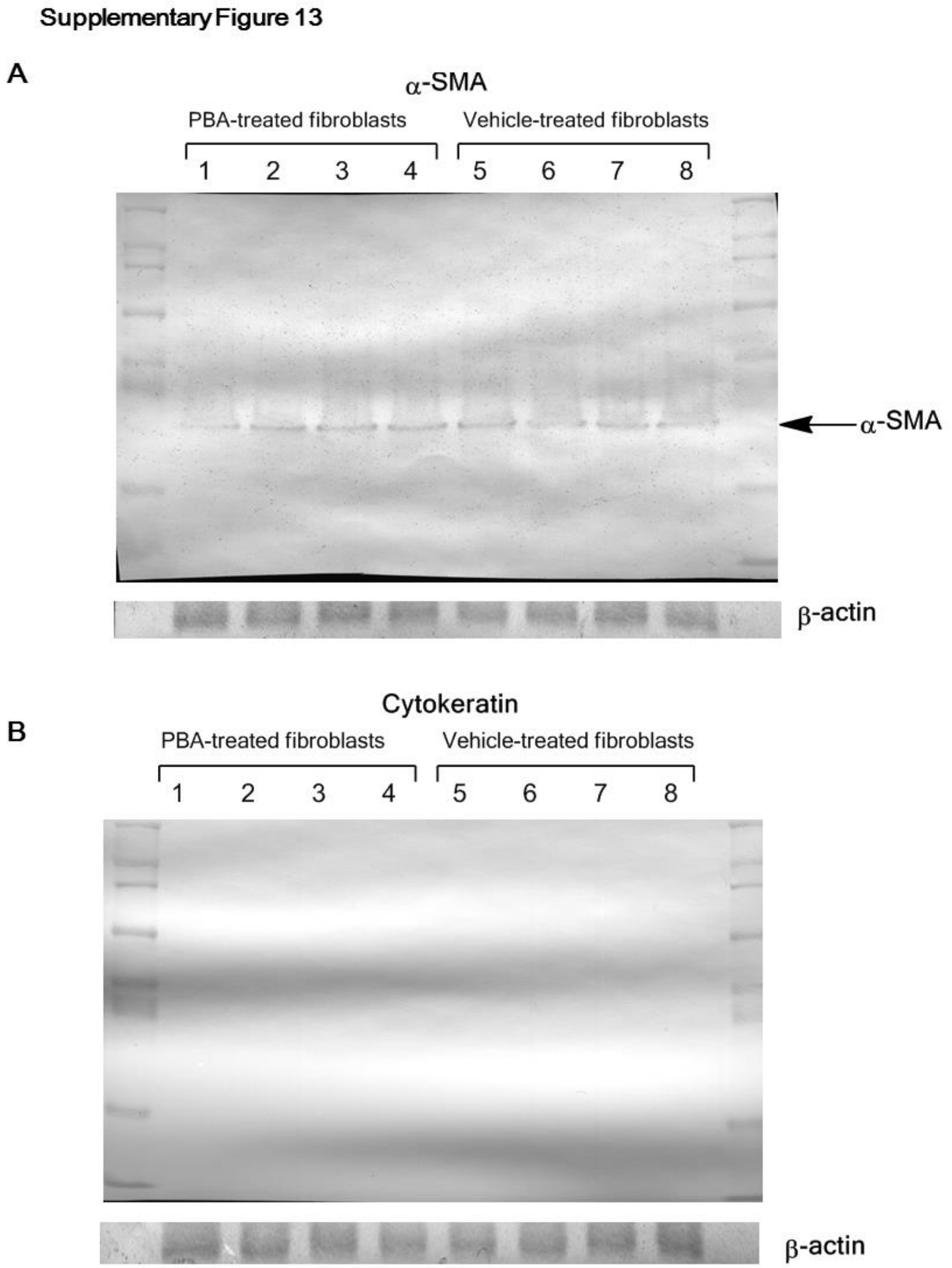

Supplementary Figure 13. Full-length gels from immunoblot assays for α-SMA and cytokeratin. (Lanes 1, 2, 3, 4: Fibroblasts from the PBA-treated lacrimal glands, Lanes 5, 6, 7, 8: Fibroblasts from the vehicle-treated lacrimal glands). (A) α-SMA, (B) cytokeratin

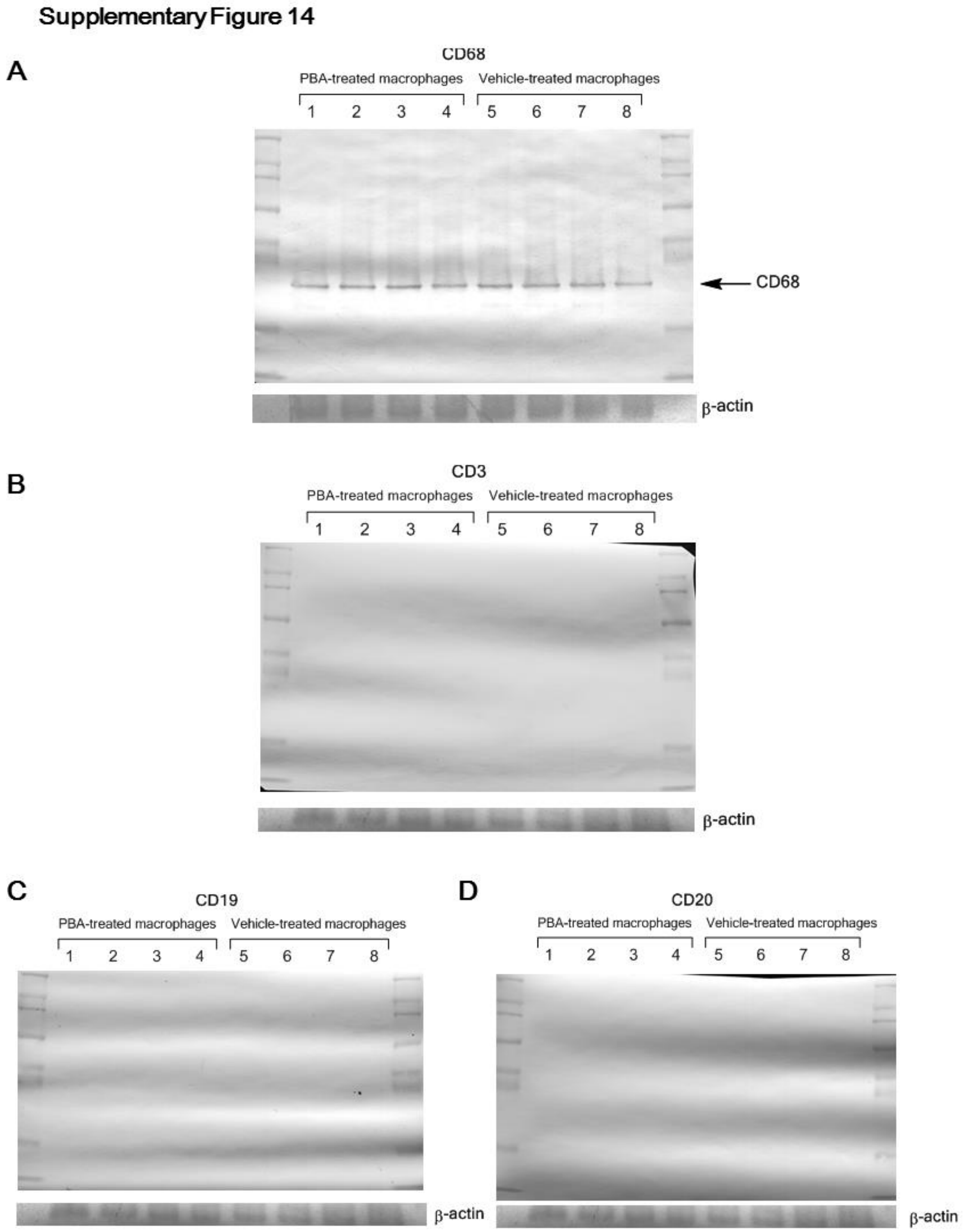

Supplementary Figure 14. Full-length gels from immunoblot assays for CD68, CD3, CD19 and CD20. Lanes 1, 2, 3, 4: Splenic macrophages from PBA-dosed mice, Lanes 5, 6, 7, 8: Splenic macrophages from vehicle-dosed mice). (A) CD68, (B) CD3, (C) CD19, (D) CD20

References

1. Hopwood, J. Fixation and fixtative. In: Bancroft JD, Stevens A, eds. *Theory and Practice of Histological Techniques. 4th ed. Edinburgh: Churchill–Livingstone* 23-46 (1996).

2. Anderson, G. & Gordon, K. Tissue processing, microtomy and paraffin sections. In: Bancroft JD, Stevens A, eds. T. *Theory and Practice of Histological Techniques. 4th ed. Edinburgh: Churchill–Livingstone*, 47-68 (1996).
